# Evidence for Small RNAs Homologous to Effector-Encoding Genes and Transposable Elements in the Oomycete *Phytophthora infestans*

**DOI:** 10.1371/journal.pone.0051399

**Published:** 2012-12-14

**Authors:** Ramesh R. Vetukuri, Anna K. M. Åsman, Christian Tellgren-Roth, Sultana N. Jahan, Johan Reimegård, Johan Fogelqvist, Eugene Savenkov, Fredrik Söderbom, Anna O. Avrova, Stephen C. Whisson, Christina Dixelius

**Affiliations:** 1 Department of Plant Biology and Forest Genetics, Uppsala BioCenter, Swedish University of Agricultural Sciences and Linnean Center for Plant Biology, Uppsala, Sweden; 2 Department of Immunology, Genetics and Pathology, SciLifeLab, Rudbeck Laboratory, Uppsala University, Uppsala, Sweden; 3 School of Biotechnology, KTH, SciLife Laboratory, Solna, Sweden; 4 Department of Cell and Molecular Biology, Biomedical Center, Uppsala University, Uppsala, Sweden; 5 Cell and Molecular Sciences, The James Hutton Institute, Invergowrie, Dundee, United Kingdom; University of Wisconsin-Milwaukee, United States of America

## Abstract

*Phytophthora infestans* is the oomycete pathogen responsible for the devastating late blight disease on potato and tomato. There is presently an intense research focus on the role(s) of effectors in promoting late blight disease development. However, little is known about how they are regulated, or how diversity in their expression may be generated among different isolates. Here we present data from investigation of RNA silencing processes, characterized by non-coding small RNA molecules (sRNA) of 19–40 nt. From deep sequencing of sRNAs we have identified sRNAs matching numerous RxLR and Crinkler (CRN) effector protein genes in two isolates differing in pathogenicity. Effector gene-derived sRNAs were present in both isolates, but exhibited marked differences in abundance, especially for CRN effectors. Small RNAs in *P. infestans* grouped into three clear size classes of 21, 25/26 and 32 nt. Small RNAs from all size classes mapped to RxLR effector genes, but notably 21 nt sRNAs were the predominant size class mapping to CRN effector genes. Some effector genes, such as *PiAvr3a*, to which sRNAs were found, also exhibited differences in transcript accumulation between the two isolates. The *P. infestans* genome is rich in transposable elements, and the majority of sRNAs of all size classes mapped to these sequences, predominantly to long terminal repeat (LTR) retrotransposons. RNA silencing of Dicer and Argonaute genes provided evidence that generation of 21 nt sRNAs is Dicer-dependent, while accumulation of longer sRNAs was impacted by silencing of Argonaute genes. Additionally, we identified six microRNA (miRNA) candidates from our sequencing data, their precursor sequences from the genome sequence, and target mRNAs. These miRNA candidates have features characteristic of both plant and metazoan miRNAs.

## Introduction

Small noncoding RNAs (sRNAs) of 20–40 nucleotides (nt) have emerged as key players in both transcriptional and post-transcriptional gene regulation in an array of organisms [1]. They also play important roles in genome stability, chromatin organization, and virus resistance [2]. Since their discovery, diverse classes of sRNAs have been described, and new types of sRNAs and pathways continue to be reported. The most commonly referred to are microRNAs (miRNAs), small interfering RNAs (siRNAs), and Piwi-interacting RNAs (piRNAs), based on their biogenesis and Argonaute (AGO) protein-binding partners [3], [4]. The biogenesis and function of sRNAs typically involves a core set of proteins, namely Dicer or Dicer-like (DCR or DCL; double stranded (ds)RNA ribonuclease), Argonaute (AGO; RNA binding, slicer ribonuclease), and RNA-dependent RNA polymerase (RdR; secondary sRNA production).

Oomycetes, also known as water molds, are fungus-like heterotrophs which are ubiquitous in marine, freshwater and terrestrial environments [5]. This group of organisms belongs to the lineage of biflagellate Stramenopiles within the kingdom Chromista [6], [7]. Brown algae and diatoms are also grouped within the Stramenopiles, and thus oomycetes share an evolutionary history with photosynthetic organisms [8]. Oomycetes are also distantly related to the Apicomplexa, such as *Plasmodium* and *Toxoplasma* parasites [9].

*Phytophthora infestans* is a destructive plant pathogen best known for its role in the mid-19^th^ century Irish potato famine. This oomycete plant pathogen remains a threat to food security due to its impact on the world’s fourth most important food crop, potato. It also infects tomato, another globally significant crop. The lifecycle of *P. infestans* involves both asexual and sexual reproduction pathways (Figure S1). Spore dispersal is mainly via asexual sporangia that can germinate directly within a few hours after landing on potato or tomato foliage. Alternatively, sporangia can release motile biflagellate zoospores that are attracted to potential plant hosts and new infection sites. Both propagule dispersal mechanisms lead to rapidly spreading disease epidemics. Further, this plant pathogen secretes numerous proteins (effectors) that are important for colonization and promotion of disease (pathogenicity) on its host plants. The diverse effector repertoire of *P. infestans* assists pathogen adaptation to a changing host environment, leading to frequent breakdown of introduced resistance in crop plants [10], [11]. The most studied oomycete effectors are the RxLR and Crinkler (CRN) groups that are delivered to the host cytoplasm and are characterized by conserved peptide motifs required for translocation into the host [12], [13].

The genome size of *P. infestans* is two- to four-fold greater than those of the other sequenced plant pathogenic *Phytophthora* species, *P. sojae*, *P. ramorum* [14], and *P. capsici* [15]. One reason for the increased size of the *P. infestans* genome (240 Mb) is the large number of transposable elements (TEs), chiefly retrotransposons, and repeated sequences. The *P. infestans* genome is organized into gene-rich regions that contain relatively little repetitive DNA, and gene-poor regions that are enriched for repetitive DNA that includes a high proportion of transposons [14]. The repeat-rich regions are also enriched for effector-encoding genes. It has been proposed that repeats may be associated with pathogenicity effector family expansion and genome reorganizations [14], [16], [17].

Gene silencing is known to occur in oomycetes, and this has been best studied in *P. infestans*, for which it has been exploited to determine the role(s) of specific genes in the lifecycle or during infection [18]–[20]. However, despite being the most intensely studied oomycete, the molecular mechanisms underlying gene silencing in *P. infestans* are not as well characterized as in other model eukaryotes. Expression of sense and antisense gene constructs, followed by nuclear run-on assays, suggests the involvement of transcriptional gene silencing processes [21]. This is most likely mediated through heterochomatin formation that relies on histone modifications such as deacetylation and methylation, but does not involve cytosine methylation [21], [22]. The formation of heterochromatin may spread for short distances (hundreds of bp) away from the silenced region. In contrast, treatment of protoplasts with dsRNA results in transient RNA silencing, and was proposed to be initiated via the post-transcriptional silencing machinery [23], [24].

While much of the focus in understanding RNA silencing has been directed towards mouse, zebrafish, *Caenorhabditis elegans*, *Drosophila melanogaster, Arabidopsis thaliana*, and *Schizosaccharomyces pombe*, comparatively few reports exist from the phylogenetic branch that includes the Stramenopiles and Apicomplexans. RNA silencing pathways in these unicellular eukaryotes have revealed some striking differences to several model organisms, such as atypical DCL proteins and unusual biogenesis of miRNAs [25], [26]. To advance the understanding of gene silencing processes operating in *P. infestans*, we have previously shown that *P. infestans* possesses the components of canonical gene silencing pathways similar to those of other eukaryotes [27]. However, genes encoding silencing-related proteins such as cytosine methyltransferases, RNA polymerase IV, Drosha and ERI1, present in other organisms, were not identified from its genome sequence.

In this study we used deep sequencing (SOLiD) to characterize classes of sRNAs from two *P. infestans* isolates with contrasting virulence and pathogenicity. We determined the relative abundance and distribution of sRNAs in the predicted transcriptome, effector genes, and TEs. Of particular interest are questions regarding how *P. infestans* controls the excessive transposon load that has profoundly shaped its genome, and how traits such as pathogenicity and specific virulence/avirulence may connect to variations in the presence or prevalence of sRNAs. Our data revealed the expected 21 nt sRNAs, but new sRNA size classes of 25/26, 31/32, 35 and 40 nt were also identified. Most commonly, sRNAs were derived from TEs, followed by CRN and RxLR effector genes. Generation of 21 nt sRNAs involves PiDcl1 (Dicer-like), as revealed by analysis in *P. infestans* lines silenced for PiDcl1. Furthermore miRNA sequences were predicted by combining sRNA sequencing with precursor prediction from the genome sequence.

## Results and Discussion

### *P. infestans* Produces at Least Three Size Classes of sRNA Molecules

Isolate R0 is weakly pathogenic on potato plants lacking any known resistance genes. In contrast, isolate 3928A is highly pathogenic, and exhibits specific (gene-for-gene) virulence on potato plants carrying the *R1–R7*, and *R10–R11* resistance genes [28]. Compared to isolate 3928A, R0 produces relatively few sporangia, which in turn produce relatively few zoospores (and subsequent cysts). From both isolates, sRNA fractions were prepared from cultured non-sporulating mycelia, sporangia, germinating sporangia, and germinating cysts. Sequencing of sRNAs from eight SOLiD libraries generated a combined total of 12.8 million sequence reads for 3928A and 15.3 million for R0 (Table S1). Small RNAs that were derived from tRNAs and rRNAs, representing 2–4% of total sequence reads, were filtered out before mapping to other datasets. To assure that the sRNA sequences were not simply the products of mRNA turnover, they were first mapped to a small set of highly expressed single copy number genes that included (as examples) actin, ß-tubulin, and ubiquitin from *P. infestans*. Consistent with other studies that have used ABI SOLiD sequencing of sRNAs [26], a large proportion of the remaining sequence reads did not match any of the genomic sequences and were discarded. A high proportion of unmapped sRNAs has been reported previously from SOLiD sequencing experiments and may be attributable to the level of filtering applied [29]. That is, SOLiD reads are reported immediately without filtering, while other sequencing technologies such as 454 and Illumina incorporate a pre-filtering step. Of the sRNAs not derived from tRNAs and rRNAs, 6.6–15.6% mapped to the dataset of the entire genome sequence (core set), and a further 6.2–17.0% mapped to the dataset of unplaced sequence reads from the *P. infestans* genome database (www.broadinstitute.org) (Table S1; Figure S2A, B). The exact composition of the unplaced sequence reads is not well defined, but is likely to contain highly repetitive sequences that could not be accurately placed in the assembled *P. infestans* genome. Of the core set, 46.2–87.3% were homologous to the total predicted transposons, 14.5–23.5% mapped to the total predicted mRNAs, 0.1–0.3% to RxLR and 0.1–1.9% to CRN effector genes (Table S1).

Alignment of all sRNA sequences in the core set to the entire *P. infestans* genome sequence revealed an enrichment for size classes of 21 and 25/26 nt (Figure S2A). Although not forming a clear peak in the histogram, sRNAs in the range of 31–33 nt were also prevalent. Indeed, sRNAs of 32 nt were the most abundant size class in the R0 isolate. All of the sRNA libraries sequenced displayed a similar size distribution of sRNAs. The exceptions to these were the sporangia and germinating cysts libraries from R0. These showed relatively low abundance of 21 and 25/26 nt sRNAs, and enrichment for 31–33 nt sRNAs (data not shown). The existence of multiple sRNA size classes in *P. infestans* was in contrast to findings from organisms such as *D. melanogaster*, *Dictyostelium discoideum, Toxoplasma gondii*, and *Cryptococcus neoformans*, which exhibited marked abundance of a single sRNA length [25], [30]–[32]. In the diatom *Thalassiosira pseudonana*, SOLiD sequencing of sRNA identified enrichment for 22 nt sRNAs, while longer sRNAs were identified by 454 sequencing [26]. Similarly, in members of the “green lineage”, such as the moss *Physcomitrella patens*, and higher plants *Populus trichocarpa* and *A. thaliana,* more than one size class of sRNA is also prevalent [33]–[35].

### Abundant sRNAs Derived from CRN Effector Genes and Long Terminal Repeat (LTR) Retro-transposons

We aimed to determine if mapping of sRNA sequences to the specific sequence datasets of TEs and effector genes (RxLRs, CRNs), could reveal a differential distribution of matching sRNAs. We focused on these three categories since the expression of *P. infestans* effectors is considered to be the main determinant of pathogenicity [36], and *P. infestans* has the most transposon-rich genome of the oomycetes [14]. Transcripts from TEs have also been identified previously among *P. infestans* expressed sequence tags and in microarray hybridizations [14], [37], [38]. Furthermore, over half of the RxLR effector genes predicted from the *P. infestans* genome are within 2 kb of transposon-derived sequences, and it has been hypothesized that RNA silencing of transposons may also influence expression of nearby RxLR effectors through formation of heterochromatin [17], [39].

**Figure 1:**
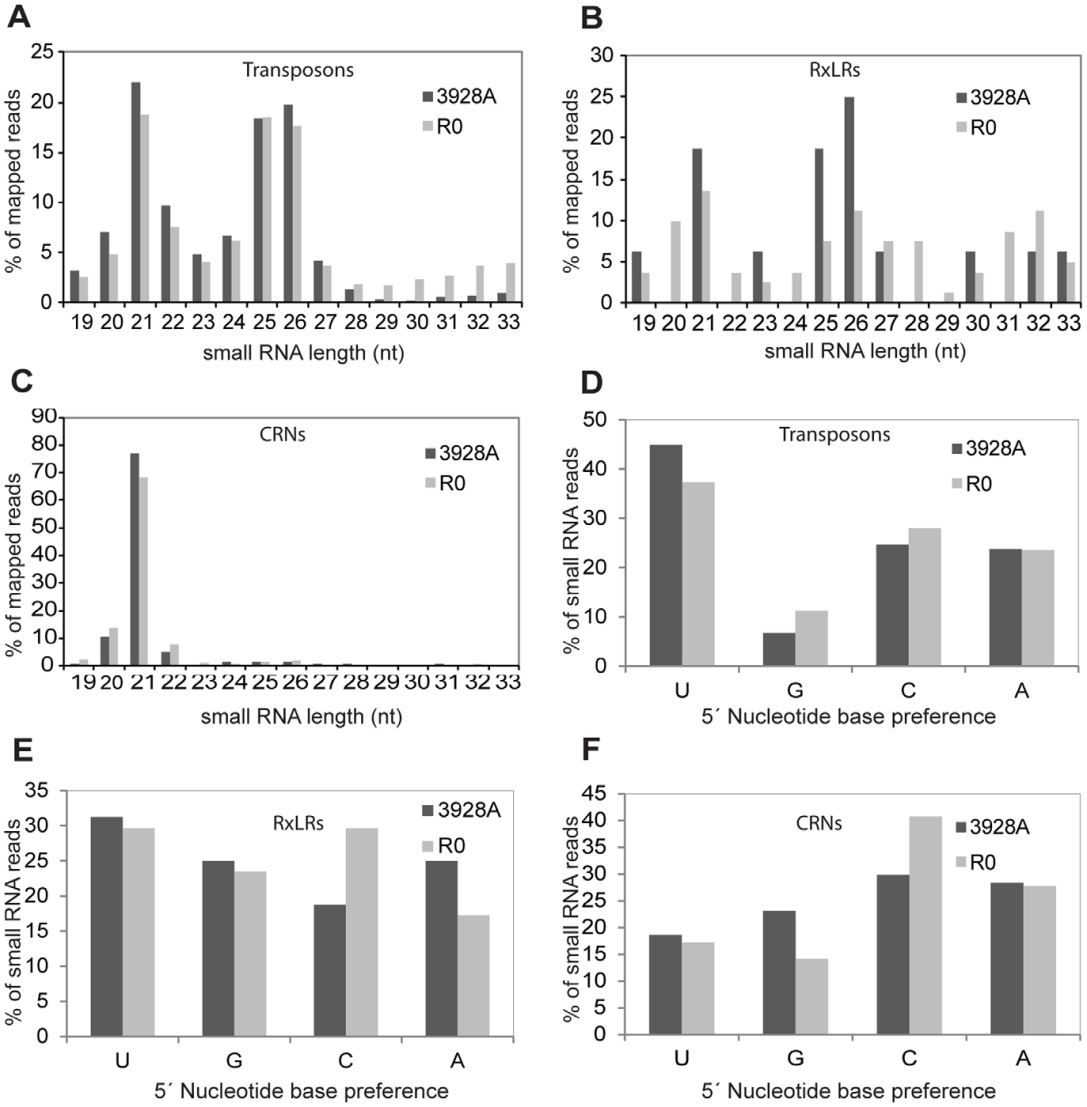
Size distribution and 5′ nucleotide preferences of sRNAs mapped to transposons, RxLR and CRN effector gene subsets in *P. infestans* isolates R0 and 3928A. Abundance of each size class of sRNAs based on nucleotide (nt) length in: **A**. Transposons **B**. RxLRs and **C**. CRNs. The relative frequency of each 5′ terminal nucleotide of sRNAs aligned to: **D**. Transposons **E**. RxLRs and **F**. CRNs.

An overwhelming majority of 21 nt sequences was the most striking feature of sRNAs derived from CRN genes (67–77% of all CRN-mapping sRNAs; Figure 1C). In comparison, sRNAs mapped to TEs were most prevalent at sizes of 21 (17–22%), 25/26 (35–38%), and 31–33 nt (3–11%) (Figure 1A). Small RNAs mapped to RxLR effector genes also exhibited enrichment in specific size classes, although the distribution was not as narrow as for CRN effector genes (Figure 1B, C). Small RNAs mapping to the unplaced sequence reads from the *P. infestans* genome database (www.broadinstitute.org) revealed a marked enrichment (28%) of 32 nt sRNAs (Figure S2B) in the R0 isolate, similar to that found for sRNAs mapped to the entire genome (Figure S2A). The R0 isolate also exhibited a higher proportion of 31–33 nt sRNAs derived from the TE (11%) and RxLR (23%) categories (Figure 1A, B).

We assessed the comparative abundance of sRNAs derived from each RxLR, CRN, and transposon group in all of the libraries from 3928A and R0 (Table S2). For the majority of transposon or effector gene sequences, the difference in sRNA abundance was no more than two-fold between the two isolates for comparable libraries (for example R0-mycelium versus 3928A-mycelium). The maximum difference for transposon-derived sRNAs was observed in the germinating cysts libraries, where sRNAs from two different novel *PiggyBAC* transposons were 5-fold to 9-fold more abundant in 3928A compared to R0, respectively. The greatest difference for sRNAs derived from RxLR and CRN effector encoding genes was found between the germinating cyst and sporangia libraries for the two isolates. Here, sRNAs derived from CRN (*PITG_18133*) were found to be 84-fold more abundant in 3928A. The CRN effectors have been proposed to represent an ancient class of pathogenicity effector proteins that have evolved through recombination among gene family members, and many CRN gene copies exist as pseudogenes or have been subject to transposon insertion [14]. However, little is known about how they are regulated, or how diversity in their expression may be generated among different *P. infestans* genotypes. Our finding of significant accumulation of sRNAs to this effector class may be the first indication for involvement of silencing processes in CRN gene regulation.

The fact that the majority of sRNAs were homologous to TE sequences was not surprising since the *P. infestans* genome is predominantly composed of these elements. The *P. infestans* genome hosts a wide diversity of highly repeated transposons including: *Gypsy* and *Copia* LTR retrotransposons, *Cryptons*, *Helitrons*, *DIRS*, *hAT*, *Mutator*, *Mariner*, *Dodo*, *Piggy-BAC*, *Pogo*, and other novel DNA transposons and LTR retrotransposons [14]. To determine if the class of TE resulted in a specific sRNA size distribution or sense/antisense orientation, sRNA sequences were matched to datasets of specific groups of TEs (Figure S3, S4). The majority of sRNAs in our datasets were derived from LTR retrotransposons (Figure S3B); these are the most prevalent class of TEs in *P. infestans* and account for over 30% of the entire genome [14]. LTR retrotransposons were distinguished by an approximately two-fold greater abundance of 21 nt sRNAs, compared to 25/26 nt sRNAs (Figure S3B). Conversely, *Mutator* and *Mariner* DNA transposons (Figure S3H, I) showed an approximately two-fold greater abundance of homologous 25/26 nt sRNAs, compared to 21 nt. However, the most striking example of sRNA size bias was observed for *HelENtrons* (endonuclease-containing *Helitron*). The sRNA size distribution for this class of TEs was most similar to that observed for CRN effector genes, with an approximately ten-fold bias towards 21 nt sRNAs, compared to 25/26 nt sRNAs (Figure S3N).

DCR digestion of long dsRNA yields duplex sRNAs, and typically only the antisense strand (complementary to the target transcript) is incorporated into the AGO-containing RNA-induced silencing complex (RISC), and the sense strand is degraded [40]. Thus, an excess of antisense-aligning sRNAs may be expected when sequencing of sRNAs is performed. To determine if such bias was evident in our sRNA core dataset, we separated the sRNA read sequences into those aligning in sense and antisense directions. For some TE sequences, very few sRNAs were identified, and in these instances it is difficult to draw any clear conclusions about bias in sRNA orientation. For TEs and genes where sRNAs were commonly detected, there was no obvious bias toward antisense sRNAs in our data (Figure S4). This suggests that process(es) other than the typical dsRNA→DCL→AGO pathway may be operating in *P. infestans*.

### Small RNAs are Preferentially Derived from Specific Sites in TEs and Effector-encoding Genes

To determine if sRNAs were derived from specific sequence regions, a selection of TEs and genes encoding RxLR and CRN effectors with a high abundance of homologous sRNAs were analyzed in more detail. *Gypsy Pi-1a* and a novel *Gypsy* LTR element called ‘*Albatross*’ are the two most abundant TEs and combined, account for 29% of the *P. infestans* genome [14]. Small RNAs mapped throughout the length of these two TE classes in both sense and antisense orientations in all lifecycle stages (Figure S5A, B), with notable ‘hotspots’ of mapping sRNAs. This distribution is consistent with mapping of sRNAs to LTR transposons in other organisms [30], [31], [41].

*Cryptons*, a unique class of transposons that use a tyrosine recombinase for transposition [42], are present in *P. infestans* and other oomycetes [14]. *Crypton6* is approximately 3.2 kb long, and sRNAs were identified that mapped across the entire length of the element at low read depth in both sense and antisense orientations. The exception to this distribution pattern was at the 3′ end (at approx. 3 kb) to which abundant antisense sRNAs were identified in all lifecycle stages sampled (Figure S5C). This specific region corresponds to a tRNA-like *infSINEp* insertion [43], for which the RNA is predicted to exhibit strong secondary structure (Figure S5D).

From our analysis, two closely related CRN genes, *PITG_09052* and *PITG_09053*, exhibited marked foci of mapped sRNAs. These two genes differ by few nucleotides that are distributed across the length of the genes, and these single nucleotide sequence differences greatly influenced the mapping of sRNAs (Figure S5E, F). For both of these predicted effector genes, 21 nt sRNAs mapped predominantly to three main sites in the 5′ half of the coding region. CRN effectors consist of recombined conserved regions [14]. However, none of the sRNA foci identified here corresponded closely with proposed sites of recombination in the two CRN effector genes.

*PITG_23226* encodes a predicted secreted RxLR effector that exhibits elevated transcript accumulation in infected potato leaves [14]. Abundant sRNAs in both sense and antisense orientations were mapped specifically to the 3′end of this gene in both isolates and all lifecycle stages (Figure S5G). Examination of the genomic DNA sequence for *PITG_23226* revealed that the 3′ end of the gene overlaps with a *Copia* LTR retrotransposon, leading to probable convergent transcription. The overlap region corresponds to the site from which the majority of the matching sRNAs were derived.

### Validation of SOLiD sRNA Sequences by Northern Hybridization

**Figure 2:**
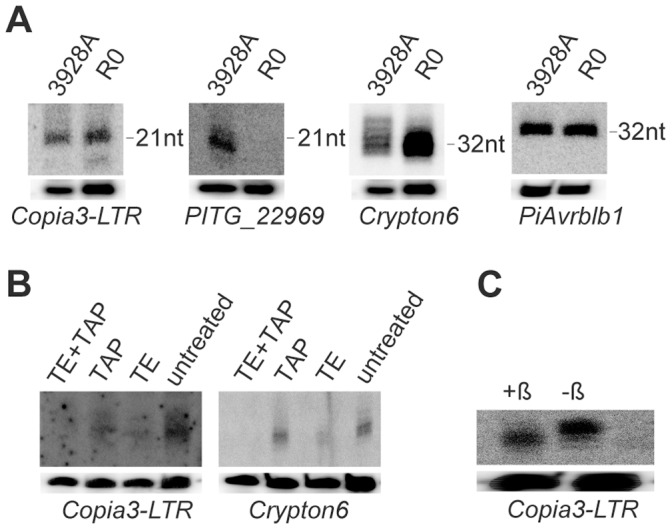
Northern hybridizations detecting *P. infestans* antisense sRNAs derived from transposons, RxLR and CRN effector genes in R0 and 3928A isolates. **A**. sRNAs hybridizing to *Copia3-LTR*, *PITG_22969* (CRN), *Crypton6*, *PiAvrblb1* (RxLR) were detected. Loading controls (U4 spliceosomal RNA) are shown below each autoradiograph. **B**. Determination of 5′ terminal modifications to sRNAs. From left, terminator exonuclease (TE) and tobacco acid pyrophosphatase (TAP) treatment of 21 and 32 nt sRNAs from *Copia 3-LTR* and *Crypton6*, respectively in isolate R0. **C**. ß-elimination assay for 3′ modifications to sRNAs, determined for 21 nt sRNAs for *Copia3-LTR* in isolate R0. Lane +β is after treatment with periodate, showing a 1 nt downward shift in size compared to the untreated sample (−β).

While our SOLiD sequencing has yielded primary insights into the sRNA populations of *P. infestans*, this technology is known to be biased towards sequences with 3′ secondary structure and a higher diversity of nucleotides near the 3′ and 5′ ends [44]. However, the same study also demonstrated that SOLiD provided a more dispersed sRNA length distribution and the most accurate estimate of sRNA abundance, compared to the two other protocols under study (Illumina versions 1 and 1.5). To confirm the existence of sequenced sRNAs, Northern blot hybridizations were employed for selected TEs and effector genes (Figure 2; Figure S6). Riboprobes or DNA oligonucleotide probes specific to *Copia3-LTR*, *Satellite2,* and *Crypton6* detected different size sRNAs for each element, ranging from 21, 26, 32, to 40 nt antisense sRNAs (Figure 2A; Figure S6V, W). Antisense and sense 35 and 40 nt sRNAs, respectively, were detected for *Gypsy Pi-1a* (Figure S6T, U).

Probing for sRNAs specific to CRN effectors *PITG_22969* and *PITG_18133* revealed the presence of the expected 21 nt antisense and sense sRNAs (Figure 2A; Figure S6R, S), but only in the highly pathogenic isolate 3928A. This agrees with SOLiD sequencing, which had identified more abundant 21 nt sRNAs in sequenced libraries from 3928A. Among RxLR effector genes analyzed, 28, 30, 32 and 35 nt sense and antisense sRNAs were detected in both isolates for *PiAvrblb1* (*PITG_21388*), *PiAvr4* (*PITG_07387*), *PiAvrblb2* (*PITG_20300*), *PiAvr3a* (*PITG_14371*), *PITG_14783*, and *PITG_16240* (Figure 2A; Figure S6A–H, K, L, O, P). Sense sRNAs of 25 and 28 nt were detected in both isolates for *PITG_06308* and *PITG_15123*, and antisense sRNAs of 26, 28, and 35 nt for *PITG_06478*, *PITG_14736*, and *PiAvr3b* (*PITG_18215*) (Figure S6I, J, M, N, Q). Small RNAs of 21 nt were not detected for any of the RxLR effector genes assessed by Northern hybridization in this study. This agrees with our sequencing results which indicated that 25/26, and 30–33 nt sRNAs were the most prevalent sizes for the RxLR effector genes selected for Northern hybridizations.

At least three size classes of 21, 25/26 and 31/32 nt were found by deep sequencing from *P. infestans*, whereas Northern blot hybridizations detected additional size classes of 35 and 40 nt that were beyond the maximum SOLiD sequence read length in this study. At the time of this study, SOLiD read lengths were comparable with other next-generation sequencing technologies such as Solexa/Illumina. Small RNAs of 21 and 40 nt have been identified previously in *P. infestans* [24], [45]. However, the identification of additional size classes of sRNA (25/26, 32, 35 nt) represents new findings in *P. infestans*. Small RNAs of 21 nt are highly prevalent in many eukaryotes [31], [46]–[49]. There have also been reports of 25/26 nt sRNAs in the distantly related apicomplexan *T. gondii,* fungi such as *Mucor circinelloides*, and plants [25], [50], [51]. These sRNAs have roles in sequence-specific mRNA degradation, systemic silencing, and methylation of homologous DNA. Longer sRNAs have been reported, which also have origins in RNA silencing [52], [53]. We will address the possible mechanism of sRNA biogenesis in *P. infestans* in a later section.

We had hypothesized that RNA silencing of effector-encoding genes, mediated by sRNAs, may contribute to the differences in pathogenicity and specific virulence against potato resistances in the two contrasting isolates analyzed here. Although sRNAs were detected by Northern blot hybridizations for all effector genes tested, differences in relative abundance of sRNAs were found between the two isolates only for some of the RxLR effector-encoding genes. Small RNAs homologous to *PiAvrblb2, PiAvr3a* and *PITG_14783* were found to be more prevalent in isolate R0 (Figure S6C–F, K, L). While sRNAs derived from *PITG_14736*, *PITG_15123*, and *PiAvr3b* were detected in both isolates, their accumulation appeared to be greater in isolate 3928A (Figure S6M, N, Q). To date, PiAvr3a and PiAvrblb2 are the only RxLR effector proteins that have been found to contribute to pathogenicity in *P. infestans* [45], [54], [55]. Our discovery of sRNAs specific to *PiAvr3a* and *PiAvrblb2* as more prevalent in the weakly pathogenic R0 isolate (Figure S7) further supports our hypothesis that RNA silencing may contribute to the variation in pathogenicity in different isolates by down-regulating specific effectors.

### Small RNAs may Affect Accumulation of Transcripts Encoding RxLR and CRN Effectors

**Figure 3:**
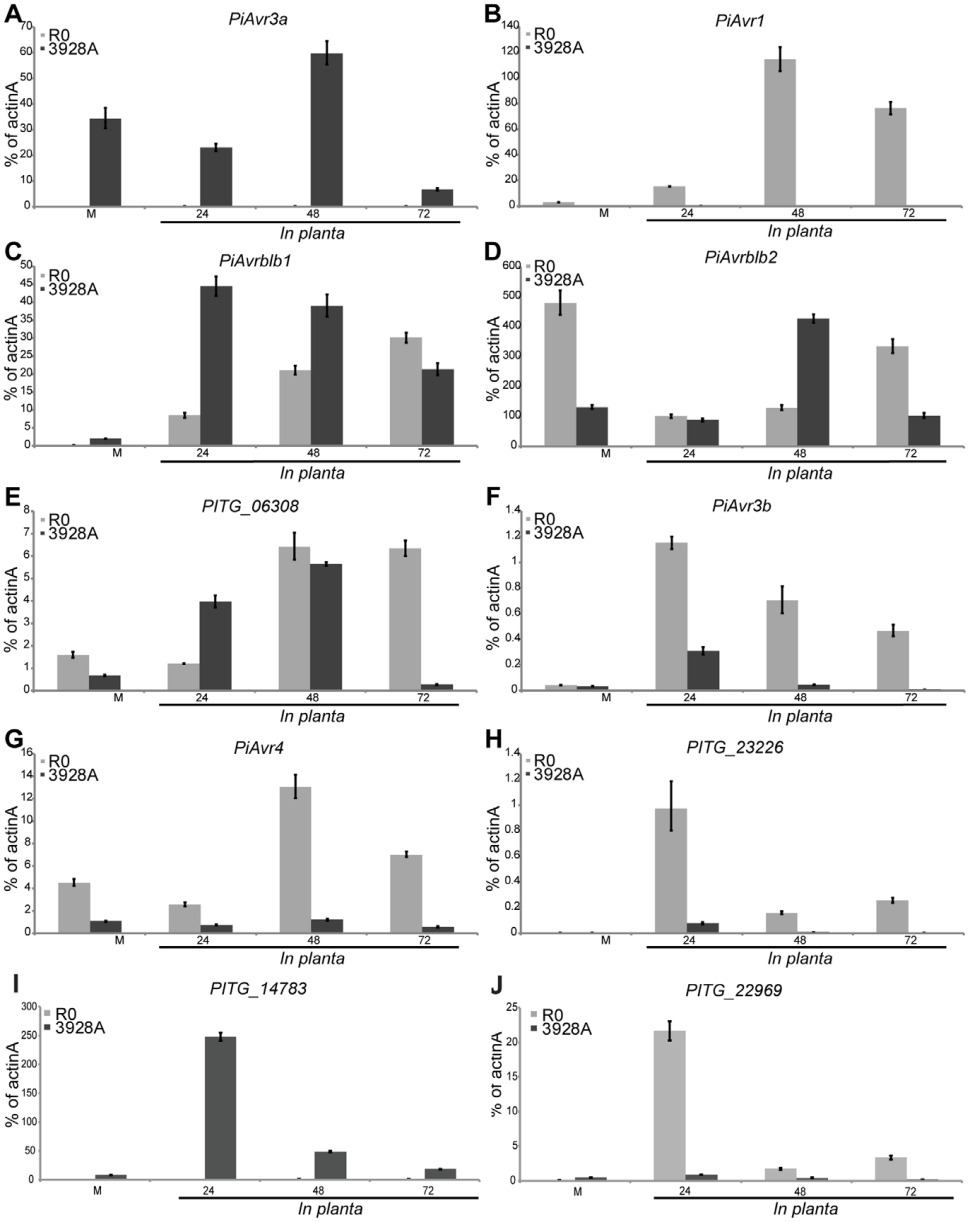
Relative transcript abundance (qRT-PCR) of RxLR and CRN effector genes at different infection time points in *P. infestans* isolates R0 and 3928A. **A**. *PiAvr3a*, **B**. *PiAvr1*, **C**. *PiAvrblb1*, **D**. *PiAvrblb2*, **E**. *PITG_06308*, **F**. *PiAvr3b*, **G**. *PiAvr4*, **H**. *PITG_23226,* **I**. *PITG_14783*, **J**. *PITG_22969*. A–I encode RxLR effectors; J encodes a CRN effector. The transcript profiles are shown at 24, 48 and 72 h post-inoculation of potato cultivar Bintje (no known resistance genes) relative to the mRNA level in cultured non-sporulating mycelium (M). In each graph, the light grey bar represents R0, and the dark bar represents 3928A. All calculations and statistical analyses were carried out as described in [90]. Error bars represent confidence intervals calculated using three technical replicates for each sample within the qRT-PCR assay. The abundance of mRNA for each gene is shown as a proportion of the *actin A* (*PiactA*) transcript on the y-axis of each graph. Amplifications repeated on independent occasions with different starting RNA and cDNA samples resulted in similar transcript accumulation profiles for all genes tested.

RxLR and other effector genes typically exhibit marked transcript accumulation during infection of plants [12], [14]. To examine if there were differences in the transcript accumulation level of RxLR effectors for which sRNAs were found, real-time (quantitative) reverse transcription polymerase chain reaction (qRT-PCR) was carried out on total RNA preparations from potato leaf samples infected with the R0 and 3928A isolates (Figure 3; Figure S8). Silencing in *P. infestans* is due to heterochromatin formation [21] and persists throughout infection of plants [20], [45], [54]. This signifies that silencing involving sRNAs *in vitro* will also be detectable during infection. First, genes encoding selected RxLR avirulence effectors were examined, since these are recognized by genetically defined major resistance proteins in potato, and act to trigger disease resistance responses. Additional effectors to which sRNAs were detected were also assessed for transcript accumulation. The *PiAvr1* gene was used as an internal control, as this gene is known to be absent from 3928A [28]. As expected, *PiAvr1* transcript was clearly detected in R0, while there was no detection of PCR products in 3928A (Figure 3B). PiAvr3a has been shown to be an essential effector for *P. infestans* pathogenicity [45], [54]. Compared to 3928A, *PiAvr3a* transcript accumulated to 227-fold lower levels in R0 (Figure 3A), consistent with sRNAs being readily identified (Figure S6E, F). In contrast, sRNAs homologous to *PiAvr3a* were either difficult to detect or undetectable in the highly pathogenic 3928A isolate (Figure S6E, F), while *PiAvr3a* transcript accumulated to high levels in this isolate. In previous experiments, silencing of *PiAvr3a* to transcript levels similar to those found here for R0 led to a loss or reduction in pathogenicity of silenced lines [45], [54]. Thus, the weakly pathogenic infection phenotype (Figure S7), accumulation of sRNAs to *PiAvr3a* (Figure S6E, F), and diminished transcript levels for *PiAvr3a* in isolate R0 (Figure 3A) are consistent with endogenous RNA silencing of this gene.

Transcripts from *PITG_14783*, *PITG_14736*, and *PITG_15123* also accumulated to significantly higher levels in 3928A compared to R0 (Figure 3I; Figure S8A, B). For *PITG_14783*, this is consistent with greater levels of homologous sRNAs in R0 (Figure S6K, L). Transcripts of *PITG_14736* accumulated to very low levels in both R0 and 3928A (Figure S8A), consistent with the identification of sRNAs for this gene in both isolates (Figure S6M). Indeed, *PITG_14736* transcripts could not be detected during any stage of infection for R0. Small RNAs were detected in both isolates for *PITG_15123* (Figure S6N), which is a member of a gene family comprised of three near-identical members [14]. It remains to be determined which *PITG_15123* gene family member is actively transcribed, or the source of homologous sRNAs.

The maximum transcript levels of *PiAvrblb1*, *PiAvrblb2* and *PITG_06308* were similar in both isolates, although the peak of transcript accumulation was delayed in isolate R0 (Figure 3C–E), reaching a maximum at 72 hpi. This delay in transcript accumulation for these genes may be due to the slow progression of colonization for the R0 isolate.

Transcripts encoding effectors *PiAvr3b*, *PiAvr4*, *PITG_23226*, *PITG_22969*, and *PITG_18133* accumulated to lower levels in 3928A than R0 (Figure 3F–H, J; Figure S8C). This was expected for *PiAvr4*, since virulent isolates possess a truncated form of this gene that is either not expressed, or only expressed at a low level [56]. *PITG_22969* and *PITG_18133* encode CRN effectors for which sRNAs were more abundant in 3928A (Figure 2A; Figure S6R, S), consistent with the markedly lower levels of these transcripts in that isolate. Isolate R0 is avirulent on plants with the *R3b* resistance gene, while isolate 3928A is virulent. The lower levels of transcript accumulation observed here for *PiAvr3b* are consistent with greater abundance of sRNAs for this gene in 3928A (Figure S6Q). Although the mechanism by which *PiAvr3b* is modified in 3928A to avoid detection by *R3b* has not been described [57], results here suggest that a reduction in transcript level may contribute to its evasion of detection. Such a mechanism has been found in the *P. sojae* system where the *PsAvr3b* avirulence effector (sequence unrelated to *PiAvr3b*) evades detection by the matching *Rps3b* resistance gene in soybean plants by a combination of sequence variation and reduced transcript accumulation [58].

The RxLR effector *PITG_23226* is part of a small gene family comprising three members (including *PITG_23014* and *PITG_23015*) that differ by few nucleotides. The qRT-PCR primer pair used for *PITG_23226* will amplify all three genes from cDNA. Considering this, the overall level of transcript accumulation is low for this gene family (Figure 3H). The overlap of the 3′ end of *PITG_23226* with a *Copia* LTR retrotransposon led to an accumulation of sRNAs across this region and may also restrict transcript accumulation for this gene and its paralogs. This is consistent with other findings showing that transcriptional fusion of a *SINE* to the *PiAvr3a* gene led to silencing of both the transgene and endogenous *PiAvr3a* transcripts [45].

Although sRNAs to all of these RxLR effector-encoding genes were identified in both R0 and 3928A, it is obvious that there are additional factors that may contribute to the level of their transcript accumulation. One consideration is that sRNAs may require accumulation to a threshold level to influence gene expression. Alternatively, many RxLR effector genes for which sRNAs were detected are present in the *P. infestans* genome as gene families comprising very closely related members [14]. From sRNA sequencing and Northern hybridizations, it is difficult to distinguish whether the sRNAs that map to RxLR families may have originated from transcriptionally active or inactive members and which paralog(s) these sRNAs may act on. *PiAvrblb2* is one such example [59] and this consideration may explain why sRNAs were readily detected for this effector in R0 (Figure S6C, D), although little difference in transcript accumulation was observed for this gene between the two isolates (Figure 3D). That is, *PiAvrblb2* transcripts may originate from gene copies that are not influenced by sRNAs.

### Small RNAs in *P. infestans* have Unmodified Termini and Exhibit Preferences for Terminal Nucleotides

Small RNAs often exhibit specific nucleotide preferences or chemical modifications that are characteristic of the pathway involved in their processing and sorting by AGO proteins. This may be observed as a preference for the 5′ terminal base, which in numerous organisms has been determined as uracil [31], [32], [41]. Although the terminal nucleotide of the siRNA has a negligible effect on AGO sorting in flies and mammals, it has a major impact on sorting in plants [4], [60]. Analysis of the 5′ base of all *P. infestans* sRNA sequences mapping to TEs showed uracil to be the most prevalent 5′ nucleotide compared to other datasets (Figure 1D–F; Figure S2C, D). However, when the 5′ nucleotide of each sRNA size class was examined, the 21 nt sRNAs most commonly had cytosine as the most prevalent 5′ base. This observation contrasts with the 5′ nucleotide of the 25/26 nt sRNAs, for which uracil is most prevalent (Figure S9). In *D. melanogaster*, non-miRNA 21 nt endogenous siRNAs also have a 5′ preference for cytosine, while Piwi/Aubergine/Ago3-bound sRNAs of 23–29 nt have a 5′ nucleotide preference for uracil [31].

To further study the 5′ and 3′ ends of sRNAs, we examined specific sRNAs that had been identified from our deep sequencing analysis. The assay used for 5′-end analysis of sRNAs involved digestion with terminator exonuclease, a processive 5′ to 3′ exonuclease that specifically targets and degrades RNA with a 5′-monophosphate, but not sRNAs with 5′-triphosphates. Northern hybridization to identify candidate 21 nt and 32 nt sRNAs to *Copia3-LTR* and *Crypton6*, respectively, demonstrated that these sRNAs are prone to degradation by terminator exonuclease, confirming that the 5′ ends have monophosphates (Figure 2B). As a control, the RNA was treated with tobacco acid pyrophosphatase (TAP; that converts 5′-triphosphate RNA into 5′-monophosphate) and sequential reaction with TAP followed by terminator exonuclease. No sRNA degradation was detected after TAP treatment, while the TAP and terminator exonuclease treatment led to complete sRNA degradation. These data indicate that *P. infestans* sRNAs mapped to *Copia3-LTR* and *Crypton6* are DCL or AGO dependent (5′ monophosphate) and not derived by the action of RdR (5′ triphosphate). Studies in *C. elegans* have shown that secondary siRNAs have a 5′ triphosphate and arise by unprimed synthesis by RdRs [61]–[63].

2′-O-methylation of the 3′ terminal ribose is an important modification that stabilizes sRNAs such as miRNAs, siRNAs in plants, piRNAs in animals, and siRNAs in *D. melanogaster* [64]. 2′-O-methylation acts as a protective mechanism against 3′→5′ degradation and 3′ uridylation of sRNA in various species. This modification is carried out by sRNA methyltransferase HUA ENHANCER1 (HEN1) which methylates sRNAs in *A. thaliana,* whereas its ortholog has similar function in other plants, animals and flies. There is no readily identifiable ortholog of HEN1 in *P. infestans*, although it does possess several predicted RNA methylases. Sodium periodate treatment, followed by β-elimination, was used to examine 3′ ends of *P. infestans* sRNAs, specifically for the presence of hydroxyl (−OH) groups at 2′ positions on the terminal ribose of *Copia3-LTR* sRNAs. If both hydroxyls are present, periodate acts on the hydroxyl groups, oxidizing them to an unstable dialdehyde, subsequently leading to β-elimination of the terminal nucleotide [65]. After the treatment sRNAs will be one nucleotide shorter, leading to a downward mobility shift. *Copia3-LTR* sRNAs from *P. infestans* were found to be sensitive to periodate (Figure 2C). Consistent with observations on the absence of a HEN1 ortholog from *P. infestans*, these results provide evidence that sRNAs do not have 3′ methylation. The examined sRNAs have 5′-monophosphate and 3′-OH and thus are most likely to be DCL or AGO dependent products [66].

### Knock-down of *DCL* and *AGO* Reveals their Roles in sRNA Biogenesis

A question that arises from our sequencing of sRNAs from *P. infestans* pertains to how a single DCL (PiDcl1), a DCL-like helicase (PiRnh5) and RdR (PiRdr1), and four distinct AGO proteins (PiAgo1-5) [27] can lead to such a diverse size repertoire of sRNAs. The involvement of some of these genes in maintaining gene silencing in *P. infestans* was shown previously when *PiDcl1*, *PiAgo1* and histone deacetylase *PiHda1* were transiently silenced by exogenous application of homologous dsRNA [27]. In those experiments, small numbers of lines exhibited significant silencing of the genes encoding components of the silencing pathway. Similar to *P. infestans*, the diatom *T. pseudonana* also has an atypical DCL protein and a DCL-like RNA helicase, but differs in having a single AGO protein [26]. However, the diatom genome contains a repetitive DNA content (including transposons) of approximately 2%, compared with 74% in *P. infestans* [14], [67]. Silencing of *PiDcl1*, *PiAgo1-5*, and *PiRdr1* genes was performed here in stable transformants of *P. infestans* (88069 isolate background) using inverted repeats to initiate silencing by RNA interference. Transgenic lines were recovered for most of the genes, and for each gene at least one line exhibited significant knock-down of transcript to below wild-type levels (Figure S10). The level of transcript knock-down ranged from 0.8% of wild-type levels for *PiRnh5*, to 48% for *PiAgo5*. The exceptions to this were *PiRdr* and *PiAgo3*, where no lines showed evidence of silencing. The low numbers of silenced lines, and the transcript levels, are consistent with experiments using transient silencing [27], and highlight the technically challenging nature of carrying out these experiments in this system. As may be expected for genes involved in RNA silencing, no transgenic lines showed complete silencing. The gene knock-down lines chosen for analysis of sRNAs were D1t7 and D1t8 (*PiDcl1*), D2t1 (*PiRnh5*), A1b (*PiAgo1/2*), A4a (*PiAgo4*) and A5c (*PiAgo5*). For further analysis of sRNA biogenesis, samples from the assessments when knock-down of silencing gene transcripts was most apparent were used in Northern hybridizations.

**Figure 4:**
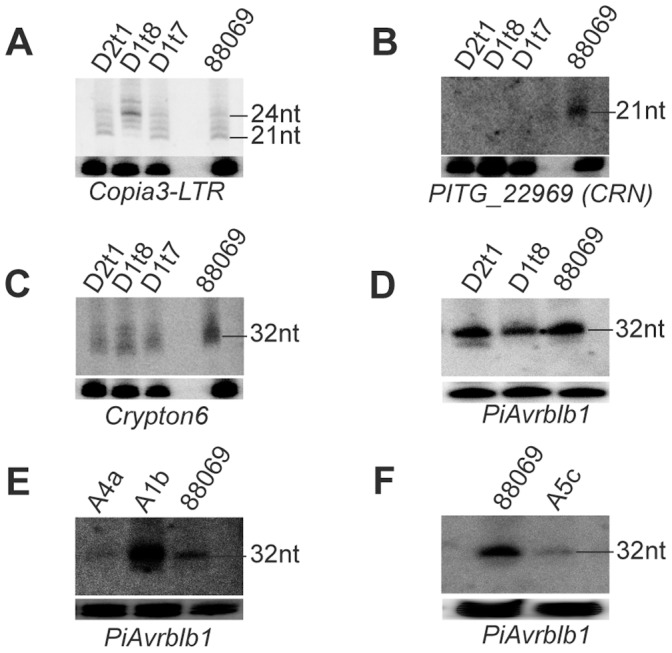
DCL-dependent generation of 21 nt sRNAs, and AGO involvement in 32 nt sRNA generation. **A**. *Copia3-LTR* 21 nt sRNAs in *PiDcl1* (D1t7, D1t8) and *PiRnh5* (D2t1) silenced lines. An upward mobility shift to approximately 24/25 nt was observed for the most silenced *PiDcl1* line, D1t8. **B**. *PITG_22969* (CRN) 21 nt sRNAs are abolished upon silencing of *PiDcl1* (D1t7, D1t8) or *PiRnh5* (D2t1). **C**. *Crypton6* 32 nt sRNAs in *PiDcl1* (D1t7, D1t8) and *PiRnh5* (D2t1) silenced lines. Silencing of these genes had no effect on the accumulation of 32 nt sRNAs. **D**. *PiAvrblb1* 32 nt sRNAs in *PiDcl1* (D1t7, D1t8) and *PiRnh5* (D2t1) silenced lines. Silencing of these genes had no effect on the accumulation of 32 nt sRNAs. **E** and **F**. *PiAvrblb1* 32 nt sRNAs in *PiAgo1*, *4*, or *5* (A1b, A4a, A5c) silenced lines. The accumulation of 32 nt sRNAs is strongly reduced in *PiAgo4* or *5* lines, while over-accumulation occurs for *PiAgo1*. Silenced lines used were *PiDcl1* (Dicer-like; D1t8, t7), *PiRnh5* (Dicer-like helicase; D2t1), *PiAgo1* (Argonaute 1; A1b) *PiAgo4* (Argonaute 4; A4a), and *PiAgo5* (Argonaute 5; A5c). Loading controls (U4 spliceosomal RNA) are shown below each autoradiograph.

The narrow distribution of sRNA sizes for CRN genes suggests a specific mechanism for generating 21 nt sRNAs. Previously in *P. infestans*, 21 nt sRNAs have only been detected in experiments where partial silencing of the *inf1* gene was observed [24]. Northern hybridizations were performed to detect 21 nt sRNAs to *Copia3-LTR* and *PITG_22969* (CRN) in *PiDcl1* and *PiRnh5* silenced lines (Figure 4A, B). Small RNAs of 21 nt from *Copia3-LTR* were absent only in *PiDcl1* line D1t8, consistent with the most pronounced reduction in *PiDcl1* transcript. Conversely, in parallel to the reduction in accumulation of 21 nt sRNAs in this line for *Copia3-LTR*, there is also accumulation of 24/25 nt sRNA. Taken together, these results suggest that, at least some 21 nt sRNAs may originate from processing of longer sRNAs that involves PiDcl1. Alternatively, another uncharacterized component of the silencing pathway is responsible for generating the 24/25 nt sRNAs homologous to *Copia3-LTR* and this enzyme would have increased substrate abundance in the *PiDcl1* silenced line.

Silencing of *PiRnh5* (DCL-like RNA helicase) indicates that, apart from PiDcl1, PiRnh5 is also required for generation of 21 nt sRNAs to *PITG_22969* (CRN) (Figure 4B). However, silencing of *PiRnh5* had no effect on accumulation of 21 nt sRNAs derived from *Copia3-LTR* (Figure 4A). This suggests that *PiRnh5* is required for silencing specific classes of genes in *P. infestans*. Studies in *C. elegans* have also shown that the helicase domain of DCR is required for accumulation of some, but not all of the endogenous siRNAs [68]. PiDcl1 has only poorly conserved partial helicase domains and no PAZ domain, and it might be speculated that PiRnh5 may act together with PiDcl1 to generate certain populations of 21 nt sRNAs. Furthermore, our results suggest that there are at least two pathways for generating 21 nt sRNAs in *P. infestans*: one pathway dependent on PiRnh5, and the other independent of PiRnh5. The mechanism by which *P. infestans* produces discretely sized and DCL-dependent 21 nt sRNA without a PAZ domain remains to be determined. The PAZ domain is considered to accurately size sRNAs during DCR processing [69], [70]. Similar to *P. infestans*, some other unicellular eukaryotes also possess DCL proteins that have no PAZ domain and may also be missing dsRNA binding or helicase domains [25], [71].

Small RNAs of 32 nt mapping to *Crypton6* or *PiAvrblb1* were unaffected by silencing of *PiDcl1* and *PiRnh5* individually, suggesting that 32 nt sRNAs are generated in DCL-independent pathways (Figure 4C, D). However, there was a clear effect on 32 nt sRNAs homologous to *PiAvrblb1* when *PiAgo* genes were silenced. Silencing of *PiAgo4* or *PiAgo5* led to a reduction in the accumulation of 32 nt sRNAs (Figure 4E, F). Surprisingly, silencing of *PiAgo1* resulted in elevated accumulation of 32 nt sRNAs (*PiAvrblb1*). Taken together, these results suggest that PiAgo4 and PiAgo5 are involved in the accumulation of 32 nt sRNAs, while PiAgo1 negatively impacts on 32 nt sRNA accumulation. Although the PiAgo proteins in *P. infestans* do not exhibit similarity to the PIWI class of proteins, the DCL-independent involvement of PiAgo4/5 in 32 nt sRNA accumulation could be speculated as analogous to the activity of PIWI in metazoans [52], [53]. Alternatively, an as yet unidentified protein with RNase activity may act to generate 32 nt sRNAs in *P. infestans*. In *S. pombe*, an AGO-dependent pathway mediates generation of the siRNAs that are indispensable for formation of pericentromeric heterochromatin, most likely through 3′–5′ trimming of Ago1-bound priRNAs by the action of the RNA exosome [72].

**Figure 5:**
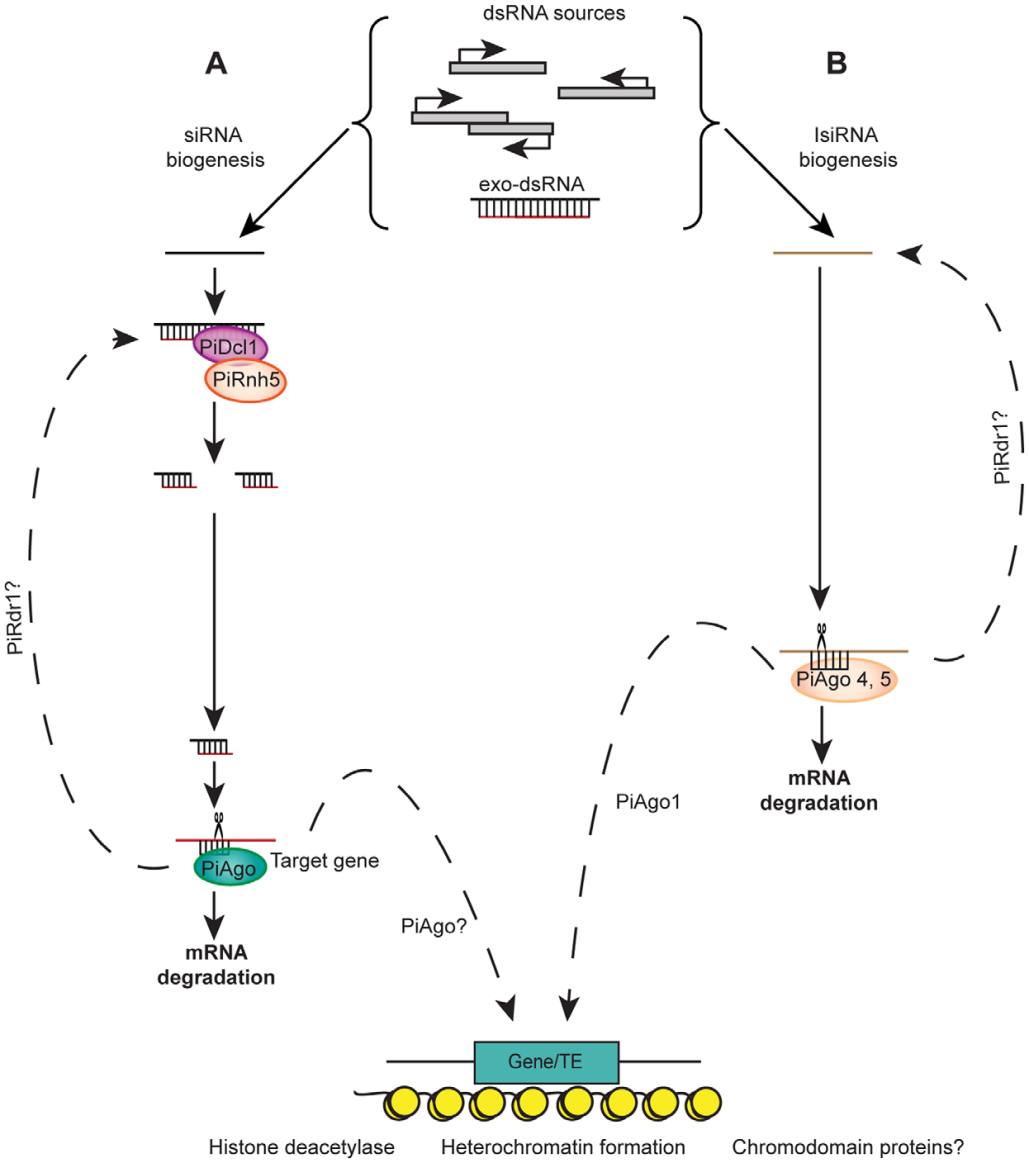
Proposed model for sRNA pathways in *P. infestans*. **A**. siRNA pathway. Transcription of repeat regions or transposons, heterochromatic regions or natural antisense transcripts and exogenous dsRNAs will lead to formation of long precursor dsRNAs. PiDcl1 processes these dsRNA into predominantly 21 nt siRNAs, in some instances in association with PiRnh5 (Dicer-like helicase). The 21 nt siRNAs generated by PiDcl1 associate with one of the PiAgo proteins, leading to degradation of target mRNAs. **B**. Long siRNA (lsiRNA) pathway. lsiRNAs are generated by PiAgo4 and 5 from transposons, coding genes, or overlapping regions of antisense transcription, or dsRNA generated by RNA polymerases. This may be a PiDcl1 independent pathway. Both pathways could be linked via one of the AGOs to heterochromatin formation aided by histone deacetylases and chromodomain proteins. RdR could further amplify the silencing.

Although sRNA pathways are generally conserved throughout plant and animal kingdoms, differences do exist between phyla. In species such as *A. thaliana, Oryza sativa* and *M. circinelloides*, there are two distinct classes of sRNA: 21 and 24/25 nt [50], [51], [73], [74]. By comparison, 21 nt sRNAs predominate in *Drosophila* [31]. The different sRNA size classes may have specific roles in RNA silencing. For example, 21 nt sRNAs from *A. thaliana* are associated with post-transcriptional silencing whereas 24 nt sRNAs are associated with transcriptional silencing [50], [75]. Earlier reports have found 21 nt sRNAs associated with partial silencing of the *P. infestans inf1* gene from a longer dsRNA precursor, but were undetectable for transcriptionally silenced lines [24]. Based on those findings, and our results here, we can suggest a model for *P. infestans* where the PiDcl1-derived 21 nt sRNAs are associated with posttranscriptional silencing, while the longer sRNAs of 32 and 25/26 nt are associated with transcriptional silencing (Figure 5).

### Prediction of miRNA Candidates, Precursors, and Targets

**Figure 6:**
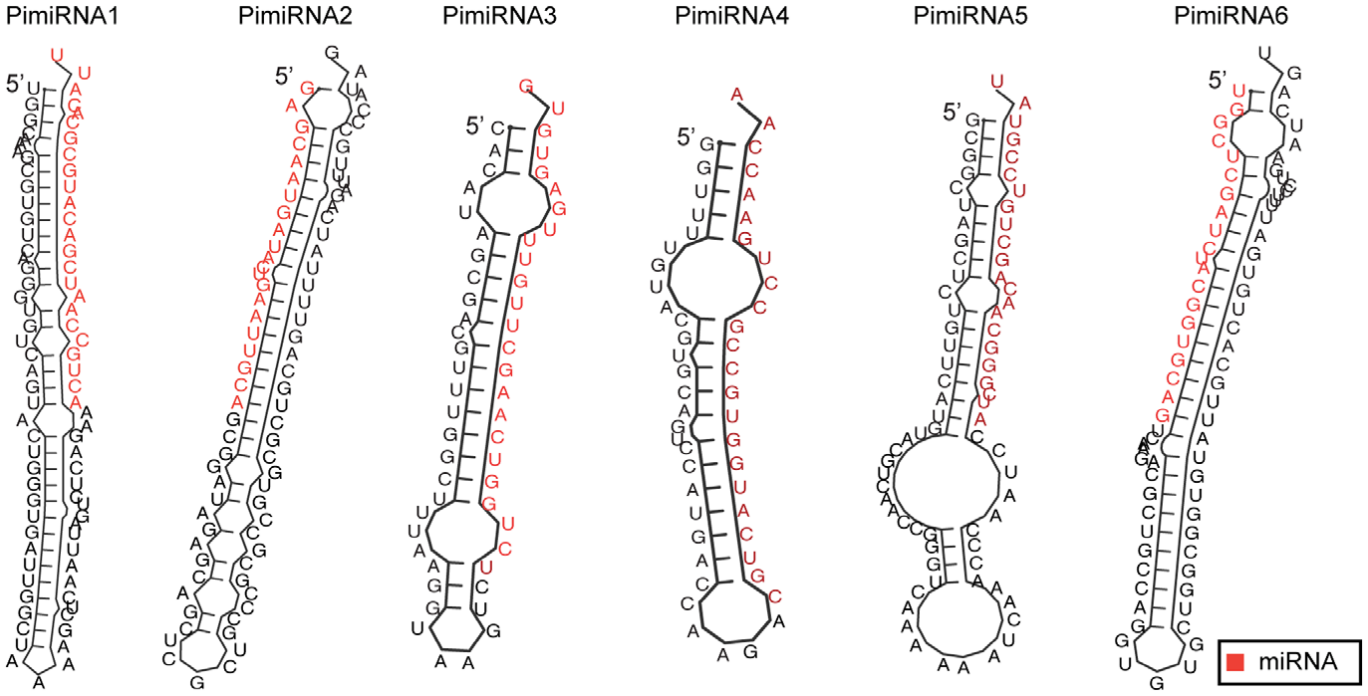
Secondary structure predictions of six miRNA candidates in *P. infestans*. Precursor miRNA sequences were folded with the RNAfold program. The miRNA sequences are shown in red.

miRNAs are abundant and major regulatory molecules that influence many essential cellular functions in a wide variety of organisms by modulating gene expression through translational repression or mRNA degradation. miRNAs have not been described from oomycetes previously, although this class of sRNA has been identified from the distantly related *T. pseudonana* [26]. We used the SOLiD sRNA sequence data to carry out a survey for putative miRNA candidates and their targets. Six putative miRNAs were predicted (Figure 6; Table S3). Occurrence of both miRNA and miRNA*(star) sequences are key features of the existence of a miRNA-miRNA* duplex and the biogenesis of miRNAs [76]. In our data we could not detect any miRNA* sequences, probably due to low abundance of star sequences in the different lifecycle stages. The size of precursor miRNA candidates ranged from 55 to 110 bp and the size of the mature miRNA candidates ranged from 21 to 24 nt (Table S3). All of the predicted mature miRNAs were present in both isolates in at least four of the eight sequenced libraries. The origin of the six predicted miRNAs varied to great extent. Two of these candidates originate from intergenic regions, while two more were found in exons, one in a 5′ untranslated region (UTR), and another in a 3′ UTR. It might be speculated that the origin of predicted miRNA candidates in *P. infestans* are more plant-like, based on the majority of candidate miRNAs originating from intergenic regions and exons [77]. These findings are echoed by studies in *T. pseudonana* and *P. patens* [26], [35]. In contrast, in animals the majority of miRNAs originate from introns [78]. We compared the predicted *P. infestans* miRNAs to the miRBase database to seek homology to known miRNAs across the entire sequence, and specifically in the seed region. No conservation with any known miRNAs was found, suggesting that these may represent a new class of miRNAs, as reported for diatoms and green algae [26].

Target prediction of candidate miRNAs used algorithms that incorporated plant-like and animal-like mapping parameters. TargetAlign prediction showed that *P. infestans* candidate miRNAs do not map to their targets with perfect complementarity. Plant miRNAs often have targets with perfect or nearly perfect complementarity, leading to target mRNA degradation [79], [80]. Conversely, animal miRNAs have relatively low complementarity to their target mRNA, suggesting translation repression [78]. Our results suggest that binding of *P. infestans* miRNA candidates to target mRNAs could occur in a manner more akin to that for animal miRNAs. Targets for *P. infestans* miRNA candidates were predominantly predicted in the UTRs and coding regions. The target mRNAs encode proteins involved in biological processes associated with nucleic acid and general metabolism (Table S4). An exception was the PimiRNA4 candidate, for which no targets were found. Studies on animal and plant miRNAs have suggested that recently evolved miRNAs may originate without pre-existing target homology and acquire targets in the course of evolution [78]. It remains possible that this may be the case for PimiRNA4.

### Conclusions

Similar to other eukaryotes, *P. infestans* produces endogenous non-coding sRNA molecules. Despite the marked differences in pathogenicity and specific virulence phenotypes for the two isolates from which sRNAs were sequenced, and the differences between the developmental stages, the sRNA populations were broadly similar. Subtle differences were identified, such as an increased abundance of 32 nt sRNAs in isolate R0, and isolate-specific increases in sRNAs derived from specific effector genes. It is likely that these differences may associate with the different infection phenotypes for these two isolates.

We have shown previously that *P. infestans* possesses DCL and AGO proteins required for RNA silencing [27]. However from the *P. infestans* RNA silencing pathway comprising single DCL and RdR proteins, and four distinct AGO proteins, it can produce an unexpectedly diverse array of prevalent sRNA sizes. This is in contrast to other systems where a single sRNA size class typically predominates. Based on former [27], [45] and present investigations in *P. infestans*, we speculate that there are at least two silencing pathways (Figure 5). One is similar to the classical DCR-dependent pathway acting on long dsRNA, processing it into 21 nt siRNAs. The other is an AGO-dependent pathway, that acts on transcripts arising from different sources and generates 25–32 nt long siRNA (lsiRNA). RdR would further amplify the silencing signal either by primer dependent or independent pathways. The siRNA and lsiRNA pathways could also be linked via one of the AGO proteins that might establish silencing via heterochromatin formation.

While the RNA silencing machinery in *P. infestans* is apparently deployed to silence the TEs that make up most of the genome, there are also many sRNAs homologous to genes encoding disease promoting effector proteins, especially CRN effectors. Endogenous RNA silencing of effector genes may contribute to variability in pathogenicity between different pathogen genotypes, such as for *PiAvr3a* in isolate R0 in this study. How silencing of these effectors is initiated, why only some effector gene paralogs may be affected, and the influence of nearby transposon sequences will be key questions in future studies directed at further understanding the influence of RNA silencing on pathogenic variation and adaptability in this economically important plant pathogen.

## Materials and Methods

### *P. infestans* Isolates

*P. infestans* isolates R0, 3928A, and 88069 were maintained at 20°C in darkness on rye agar medium amended with 2% sucrose [81], rifampicin (30 µg/ml) and pimaricin (10 µg/ml). Transgenic lines of *P. infestans* isolate 88069 were maintained as above, but agar medium was supplemented with geneticin (G418; 10 µg/ml). Isolates R0 and 3928A were used for analysis of sRNAs. R0 is a US1-like genotype [82] of A1 mating type and is weakly pathogenic only on potato containing no known resistances. 3928A is representative of the ‘Blue 13’ genotype currently dominant in British *P. infestans* populations [83]. It is highly pathogenic on potato, belongs to A2 mating type, and able to infect potato plants possessing the *R1* to *R7*, *R10,* and *R11* resistance genes [28].

### Lifecycle Stages

To prepare mycelium samples for RNA isolation, *P. infestans* was grown in liquid pea medium [23] for 5 days at 20°C, and collected by gravity filtration. Sporangia were harvested from agar plate cultures as described [84]. Germinated sporangia were prepared by allowing sporangia to germinate in sterile distilled water overnight at 20°C. Germinating cysts were prepared as described previously [84]. This sample also contained some germinating sporangia and zoospores. The different samples were snap frozen in liquid nitrogen, and stored at −70°C until used for RNA extraction.

### Pathogenicity Assessment

Susceptible potato cultivar Bintje was used to study late blight disease development for isolates R0 and 3928A. Infections were carried out as described [27]. Leaf samples were collected, snap-frozen in liquid nitrogen and stored at −70°C prior to RNA extraction.

### RNA Isolation and cDNA Synthesis

Total RNA for qRT-PCR analysis was extracted from frozen samples using the RNeasy Plant mini kit (QIAGEN) following the manufacturer’s protocol. Prior to cDNA synthesis, all RNA samples were DNase treated using the Turbo DNA-free kit (Ambion). Yield and integrity of the RNA was assessed using a NanoDrop Micro Photometer (NanoDrop Technologies), and agarose gel electrophoresis, respectively. First strand cDNA for qRT-PCR detection of *P. infestans* effector, silencing gene, and endogenous control *PiactA* transcripts were synthesized from 1 µg of total RNA by combined random hexamer and oligo dT priming using the First Strand cDNA Synthesis Kit (Quanta Biosciences) and following the manufacturer’s protocol. Total RNA for sRNA detection was prepared by TRIzol protocol (Invitrogen). The low-molecular-weight RNA was extracted using an enrichment procedure for sRNAs [85]. For Northern hybridizations, the quality of the extracted RNA was verified by ethidium bromide staining.

### SOLiD Sequencing and Computational Analysis

Total RNA for deep sequencing was prepared from the different life cycle stages, described previously, using the mirVana™ miRNA Isolation Kit (Ambion), according to the manufacturer’s protocol. The amount and quality of sRNA was estimated using an Agilent Bioanalyzer (Agilent Technologies) using settings recommended by the manufacturer. RNA library preparation and sequencing were performed using a SOLiD (version 2) platform (Applied Biosystems). Library preparation was according to the manufacturer’s recommendations, using the sRNA library protocol provided with the SOLiD Total RNASeq kit (Applied Biosystems). SOLiD sequencing generated reads that were 35 bp long. Sequence barcodes were decoded, matching each bead sequence with the identity of the sample. Sequence analysis was performed using the SOLiD System Small RNA Analysis Tool (Applied Biosystems), permitting one mismatch in the seed and a total of three mismatches over the whole read. Adaptor sequences were removed, and all sequences that matched RNAs such as tRNA or rRNA were filtered out. Only sequences of 19 to 33 nt were used in further analyses. The remaining sRNA reads were aligned independently to the total genome sequence, and to separate datasets of *P. infestan*s transposons, RxLR and CRN effectors, all predicted genes, and unplaced genome sequencing reads. Reads matching more than three locations were filtered out, except for when mapping to the whole set of transposons. All sequence datasets were downloaded from the Broad Institute (www.broadinstitute.org). Counting of sRNA lengths and starting base was done using in-house tools.

### Detection of sRNA Molecules Homologous to Transposons and Effector Genes

Cloning of effector-encoding genes and TEs for generation of riboprobes was performed as described previously [45]. Oligonucleotide primers designed for effector-encoding genes and TEs sequences are shown in Table S5. For detection of sRNAs, 20–30 µg of the sRNA fraction was separated on 12–15% polyacrylamide Tris-borate-EDTA-urea gels. RNA was transferred to nylon membrane by capillary blotting, pre-hybridized, hybridized, and washed as described [85]. The membranes were exposed in phosphorimager cassettes and scanned with a Molecular Phosphorimager FX (Molecular Dynamics).

### Plasmid Construction and Transformation

Phusion DNA polymerase (Finnzymes) was used to amplify *P. infestans* DNA using primers listed in Table S5. Cloning of amplified DNA into restriction endonuclease sites used standard protocols [86]. Insert orientation and integrity of all cloned DNA fragments were confirmed by DNA sequencing. The background plasmid vector used for all silencing constructs was pFTORA, a version of pSTORA modified by the inclusion of an *Fse*I site (http://oomyceteworld.net/plasmids/plasmids.html). For silencing, *PiDcl1*, *PiRnh5, PiAgo1-5*, and *PiRdr1* were cloned as inverted repeats into the pFTORA vector using the *Fse*I and *Sbf*I (sense direction), and *Asc*I and *Sac*II (antisense direction) restriction endonuclease sites. Transformations of *P. infestans* for all constructs were carried out using a modified polyethylene glycol-CaCl_2_–Lipofectin protocol (http://oomyceteworld.net/) [87], [88]. All the transformants were maintained on rye-sucrose agar containing 10 mg/l G418.

### RNA Secondary Structure Prediction

All RNA secondary structures were predicted using the RNA structure program, Mfold version 2.3 (http://mfold.rna.albany.edu/) [89] with default settings. The folding temperature was reset to 20°C to reflect the temperature used for growth of the *P. infestans* cultures. The RNA structures with the lowest free energy were adopted as the most likely structures.

### SYBR Green qRT-PCR Assays

Oligonucleotide primers were designed and optimized to amplify *P. infestans* sequences (Table S5) and transcript levels were analyzed as described previously [27]. Transcript abundance in transgenic lines was compared with the level of transcript in calibrator samples (mycelium of wild type isolate 88069). The calibrator samples were assigned the relative value of 1.0, to allow comparisons to be made between lines. All qRT-PCR amplifications were repeated on independent occasions with different cDNA samples. All calculations and statistical analyses were carried out as described in ABI PRISM 7700 Sequence Detection System User Bulletin #2 (Applied Biosystems) [90]. Transcript abundance in wild type R0 and 3928A was assessed as above, except that transcript abundance from effector genes was assessed in samples from infected potato leaves. In each isolate, the abundance of effector gene transcripts in each sample was calculated relative to that of the *Actin A* (GenBank AAA33749; PITG_15117) transcript, using the difference between the threshold cycle (Ct) values for both genes. The amount of effector gene transcript was expressed as a percentage of the *Actin A* transcript [27], [90]. *Actin A* was used as an endogenous control since we had previously shown that it exhibits the least variation in transcript accumulation in diverse developmental stages, compared to other transcripts tested [27].

### Analysis of sRNA 5′ and 3′ Ends

For 5′ end analysis of *P. infestans* sRNAs, 5 µg of sRNA-enriched samples were used for each reaction, separately, with terminator exonuclease, tobacco acid pyrophosphatase (TAP), and TAP followed by terminator exonuclease treatments [91]. Small RNA samples were treated with terminator exonuclease (2 U, 30°C, 1 hr), following the manufacturer’s recommendations (Epicentre). In a separate treatment, sRNAs were also incubated with TAP (20 U, 37°C, 2 h) to remove β and γ phosphates of sRNAs, leaving only the α phosphate group attached (converts 5′-triphoshphate RNA into 5′-monophosphate). After enzymatic treatment, all samples were extracted with phenol/chloroform and resolved on a 15% polyacrylamide gel. Northern blot analysis was performed using P^32^-labeled probe, to detect the sRNA of interest. Modifications at the 3′-OH termini were determined using a β-elimination assay [65]. This reaction was carried out by treatment of sRNA (20 µg) with sodium periodate (25 mM, 25°C, 10 min), followed by heating to 45°C for 90 min. Treated sRNA were separated on denaturing 15% polyacrylamide gels and membranes probed with 5′ end-labeled DNA oligonucleotides.

### Prediction of miRNA Candidates and Targets

Prediction of miRNA candidates was performed using the mirDeep2 pipeline [92], [93]. Small RNA reads of 21–24 nt that mapped to the *P. infestans* genome using SHRIMP2 [94] were used for prediction. All miRNA candidates were required to be present in at least two libraries, and have a minimum read count of ten reads for the mature sequence of the miRNA. No miRNA candidates were allowed to map in repetitive regions or to tRNA or rRNA. The folding of putative precursors was calculated using the software RNAfold [95]. The candidate miRNA sequences were also aligned to known miRNA sequences in the miRBase miRNA database [96]–[99] (www.mirbase.org). Target prediction for the candidate miRNA was calculated using the program TargetAlign [100].

## Supporting Information

Figure S1 Disease cycle of potato late blight caused by *P. infestans*. *P. infestans* mycelium from infected tubers or from germinating oospores, cysts, or sporangia spreads into shoots produced from infected or healthy tubers, causing discoloration and collapse of the host tissue. When the mycelium reaches the aerial plant parts it produces sporangiophores, which emerge through the stomata of stems and leaves and produce sporangia. When they are dispersed and reach a plant host, depending on the environmental conditions, they may germinate directly and cause new infections. Alternatively, sporangia may release motile zoospores that then encyst and germinate. The germ tube from the sporangium or cyst penetrates directly or via stomata, the mycelium grows intercellularly and intracellular haustoria are formed. Under wet or humid conditions, new sporangiophores emerge from the stomata and new infection cycles can start. In cool, moist weather new sporangia may form within four days. When the two mating types, A1 and A2 are present in the same plant tissue, fertilization can take place (sexual recombination) and oospores may be formed, providing a new source of inoculum to initiate infections. When infected plant debris fall to the ground and decompose, the oospores are released into the soil. The thick-walled oospores can over-winter in soil, in contrast to zoospores, sporangia and mycelia that are considered short-lived. In regions with mild winters, diseased volunteer potato plants are the most important source of new inoculum.
(TIF)

Figure S2 Size distribution and 5′ nucleotide preferences of sRNAs mapped to the assembled genome and unplaced genome sequencing reads (not used in genome assembly) in *P. infestans* isolates R0 and 3928A. Abundance of each size class of sRNAs based on nucleotide (nt) length in: **A**. Genome **B**. Unplaced reads. The relative frequency of 5′ terminal nucleotide for sRNAs aligned to: **C**. Genome **D**. Unplaced genome sequence reads.
(TIF)

Figure S3 Size distribution of sRNAs mapped to individual subsets of transposon and repeat classes in *P. infestans* isolates R0 and 3928A. Abundance of each size class of sRNAs based on nucleotide (nt) length in: **A**. All transposons, **B**. LTR retrotransposons, **C**. *Crypton*, **D**. *DIRS1*, **E**. *Dodo*, **F**. *Helitron*, **G**. *hAT*, **H**. *Mutator*, **I**. *Mariner,* **J**. *PiggyBAC*, **K**. *IS4*, **L**. simple repeats, **M**. *Satellite*, **N**. *helENtron*, **O**. Novel DNA transposons, **P**. *Tc1*, **Q**. *Pogo*, and **R**. Novel repeats.
(TIF)

Figure S4 Proportions of sense and antisense sRNAs mapped to different subsets of *P. infestans* transposon classes. Small RNAs are shown as the percentage of total mapped sequences in isolates R0 and 3928A. **A**. All transposons, **B**. LTR retrotransposons, **C**. *Crypton*, **D**. *DIRS1*, **E**. *Dodo*, **F**. *Helitron*, **G**. *hAT*, **H**. *helENtron*, **I**. *Mutator*, **J**. *PiggyBAC*, **K**. *IS4*, **L**. Simple repeats, **M**. *Mariner*, **N**. *Satellite*, **O**. Novel DNA transposons, **P**. *Tc1*, **Q**. *Pogo*, and **R**. Novel repeats.
(TIF)

Figure S5 Distribution of sRNAs mapped to transposons, RxLR and CRN effector genes in different life cycle stages of *P. infestans* isolates R0 and 3928A, and specific sRNAs mapped to the predicted secondary structure (mfold 2.3) of *Crypton6*. **A**. *Gypsy Pi-1a*, **B**. *Novel LTR 1b Albatross 1*, **C**. *Crypton6*, **D**. Mapping of antisense sRNA to the 3′ end of *Crypton6*, indicated in red in the secondary structure, **E**. *PITG_09053* (CRN), **F**. *PITG_09052* (CRN), **G**. *PITG_23226* (RxLR). R = isolate R0, and 3 =  isolate 3928a; M = mycelium stage, S = sporangium, G = germinating sporangium, C = germinating cysts. The *Crypton6* structure shown in **D** is the most optimal, with the lowest free energy.
(TIF)

Figure S6 Northern hybridizations detecting sRNAs derived from RxLR and CRN effector genes, and transposons in *P. infestans* isolates R0 and 3928A. **A–B**. *PiAvr4* (AS, S), **C–D**. *PiAvrblb2* (S, AS), **E–F**. *PiAvr3a* (S, AS), **G–H**. *PiAvrblb1* (S, AS), **I**. *PITG_06308* (S), **J**. *PITG_06478* (AS), **K–L**. *PITG_14783* (S, AS), **M**. *PITG_14736* (AS), **N**. *PITG_15123* (S), **O–P**. *PITG_16240* (S, AS), **Q**. *PiAvr3b* (AS), **R–S**. *PITG_18133* (CRN; AS, S), **T–U**. *Gypsy Pi-1a* (AS, S), **V**. *Copia3-LTR* (AS), **W**. *Satellite-2* (AS). S - sense strand. AS - antisense strand. Loading controls (U4 spliceosomal RNA) are shown below each autoradiograph.
(TIF)

Figure S7 Comparison of the pathogenicity of isolates R0 and 3928A during infection of detached leaflets of potato cv. Bintje. R0 exhibited minimal or no disease (A, small circles), while 3928A formed a large sporulating disease lesion (B). Leaves were photographed 5 days after inoculation.
(TIF)

Figure S8 Relative transcript abundance (qRT-PCR) of RxLR and CRN effector genes at different infection time points in *P. infestans* isolates R0 and 3928A. **A**. *PITG_14736* (RxLR), **B**. *PITG_15123* (RxLR), **C.** *PITG_18133* (CRN). The transcript profiles are shown at 24, 48 and 72 h post-inoculation on potato cultivar Bintje (no known resistance genes) relative to the mRNA level in cultured non-sporulating mycelium (M). In each graph, the light grey bar represents R0, and the dark bar represents 3928A. All calculations and statistical analyses were carried out as described in [90]. Error bars represent confidence intervals calculated using three technical replicates for each sample within the qRT-PCR assay. The abundance of mRNA for each gene is shown as a proportion of the *actin A* (*PiactA*) transcript on the y-axis of each graph. Amplifications repeated on independent occasions with different starting RNA and cDNA samples resulted in similar transcript accumulation profiles for all genes tested.
(TIF)

Figure S9 5′ nucleotide preferences of sRNAs in *P. infestans* isolates R0 and 3928A mapped to: **A–B**. All transposons, **C–D**. LTR retrotransposon subset, **E–F**. CRN effector encoding genes, **G–H**. RxLR effector encoding genes, **I–J**. All predicted mRNAs (includes RxLR and CRN effector genes). Empty columns in a panel indicate that no sRNAs of the marked size were identified.
(TIF)

Figure S10 Silencing of genes encoding components of the silencing pathway in *P. infestans*. Comparison of *PiDcl1* (dicer-like) and *PiRnh5* (dicer-like helicase), *PiAgo* (Argonaute1, 4, 5) transcript abundance (qRT-PCR), individually, in wild type (88069; wt) and inverted repeat silenced lines. **A**. D1t1–D1t14 (*PiDcl1*), **B**. D2t1–D2t3 (*PiRnh5*), **C**. A1a–A1d (*PiAgo1*), **D**. A4a–A4d (*PiAgo4*), **E**. A5a–A5c (*PiAgo5*). All calculations and statistical analyses were carried out as described in [90]. Error bars represent confidence intervals calculated using three technical replicates for each sample within the RT-PCR assay.
(TIF)

Table S1 Distribution of sRNA reads.
(DOCX)

Table S2 Global profile of sRNAs mapping to RxLRs, Crinklers and transposons, in different life cycle stages and isolates.
(XLSX)

Table S3 Predicted miRNA sequences and the location of their origin.
(DOCX)

Table S4 Predicted miRNA sequences and their targets.
(XLSX)

Table S5 List of oligonucleotide primers used for riboprobes, DNA oligonucleotide probes, qRT-PCR, and DNA cloning.
(DOCX)
