# Supplementary figures and images for: Evidence for Small RNAs Homologous to Effector-Encoding Genes and Transposable Elements in the Oomycete Phytophthora infestans
Source: PLoS One. 2012 Dec 14;7(12):e51399. doi: 10.1371/journal.pone.0051399 (PMC3522703; doi:10.1371/journal.pone.0051399)

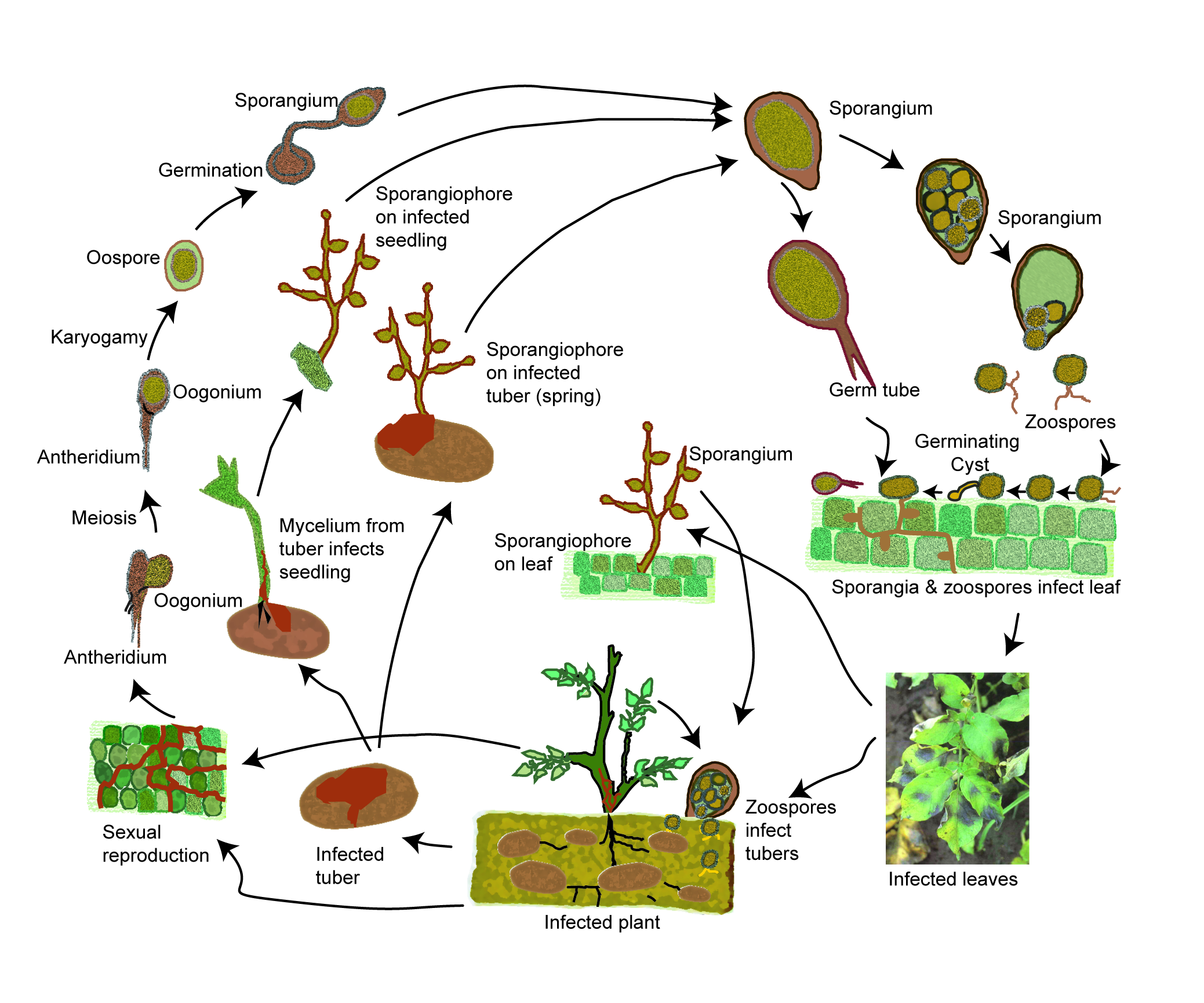

Supplement: Figure S1 — Disease cycle of potato late blight caused by P. infestans. P. infestans mycelium from infected tubers or from germinating oospores, cysts, or sporangia spreads into shoots produced from infected or healthy tubers, causing discoloration and collapse of the host tissue. When the mycelium reaches the aerial plant parts it produces sporangiophores, which emerge through the stomata of stems and leaves and produce sporangia. When they are dispersed and reach a plant host, depending on the environmental conditions, they may germinate directly and cause new infections. Alternatively, sporangia may release motile zoospores that then encyst and germinate. The germ tube from the sporangium or cyst penetrates directly or via stomata, the mycelium grows intercellularly and intracellular haustoria are formed. Under wet or humid conditions, new sporangiophores emerge from the stomata and new infection cycles can start. In cool, moist weather new sporangia may form within four days. When the two mating types, A1 and A2 are present in the same plant tissue, fertilization can take place (sexual recombination) and oospores may be formed, providing a new source of inoculum to initiate infections. When infected plant debris fall to the ground and decompose, the oospores are released into the soil. The thick-walled oospores can over-winter in soil, in contrast to zoospores, sporangia and mycelia that are considered short-lived. In regions with mild winters, diseased volunteer potato plants are the most important source of new inoculum. (TIF) [file pone.0051399.s001.tif]

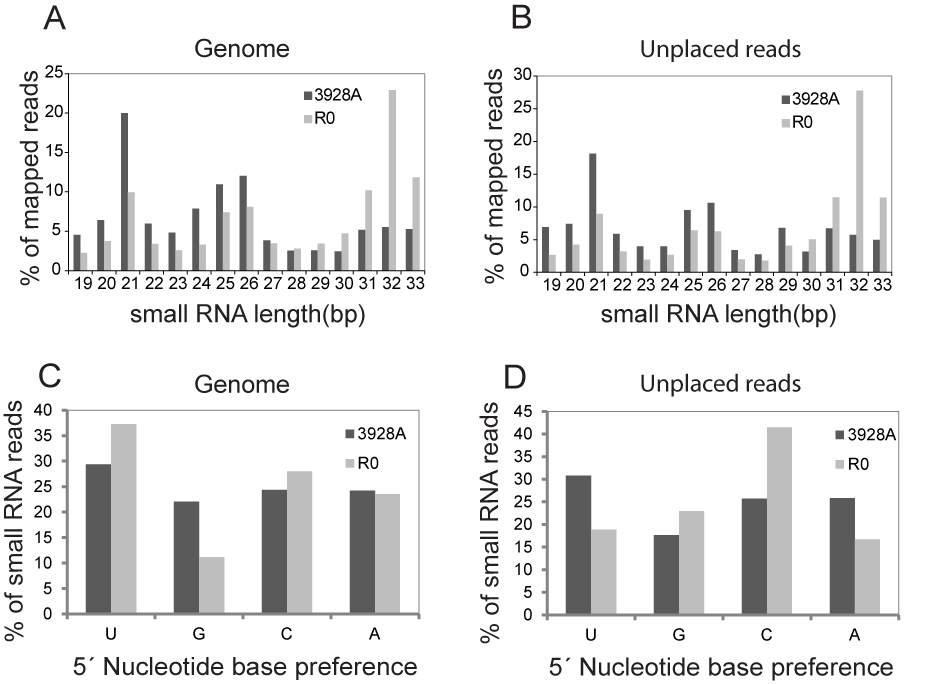

Supplement: Figure S2 — Size distribution and 5′ nucleotide preferences of sRNAs mapped to the assembled genome and unplaced genome sequencing reads (not used in genome assembly) in P. infestans isolates R0 and 3928A. Abundance of each size class of sRNAs based on nucleotide (nt) length in: A. Genome B. Unplaced reads. The relative frequency of 5′ terminal nucleotide for sRNAs aligned to: C. Genome D. Unplaced genome sequence reads. (TIF) [file pone.0051399.s002.tif]

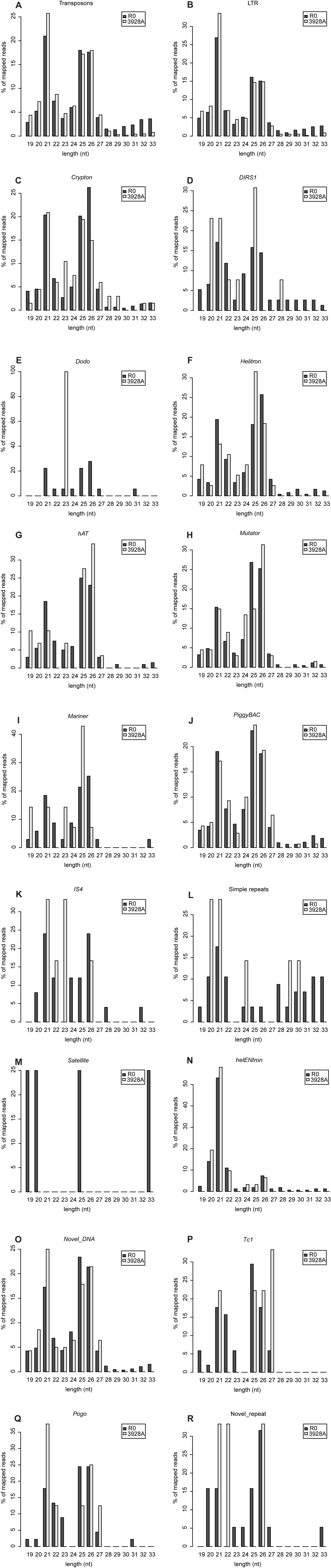

Supplement: Figure S3 — Size distribution of sRNAs mapped to individual subsets of transposon and repeat classes in P. infestans isolates R0 and 3928A. Abundance of each size class of sRNAs based on nucleotide (nt) length in: A. All transposons, B. LTR retrotransposons, C. Crypton, D. DIRS1, E. Dodo, F. Helitron, G. hAT, H. Mutator, I. Mariner, J. PiggyBAC, K. IS4, L. simple repeats, M. Satellite, N. helENtron, O. Novel DNA transposons, P. Tc1, Q. Pogo, and R. Novel repeats. (TIF) [file pone.0051399.s003.tif]

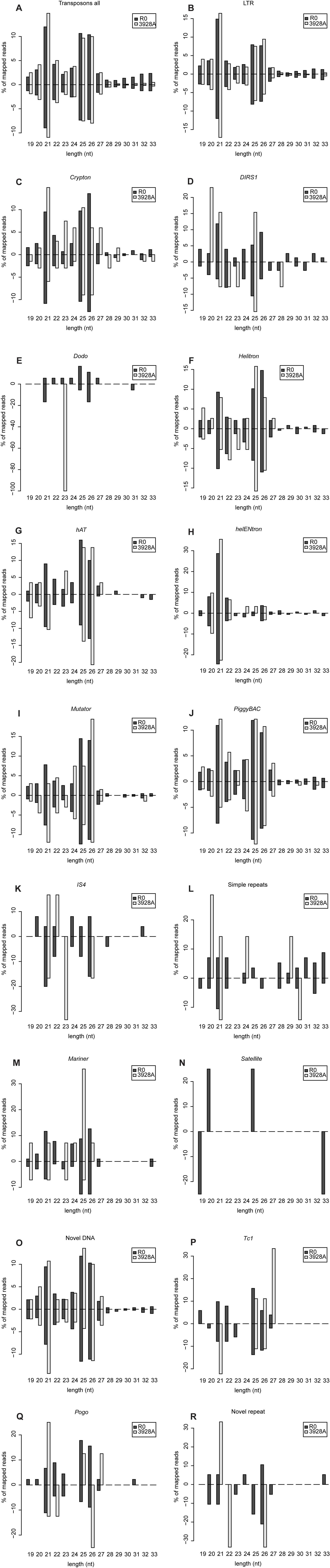

Supplement: Figure S4 — Proportions of sense and antisense sRNAs mapped to different subsets of P. infestans transposon classes. Small RNAs are shown as the percentage of total mapped sequences in isolates R0 and 3928A. A. All transposons, B. LTR retrotransposons, C. Crypton, D. DIRS1, E. Dodo, F. Helitron, G. hAT, H. helENtron, I. Mutator, J. PiggyBAC, K. IS4, L. Simple repeats, M. Mariner, N. Satellite, O. Novel DNA transposons, P. Tc1, Q. Pogo, and R. Novel repeats. (TIF) [file pone.0051399.s004.tif]

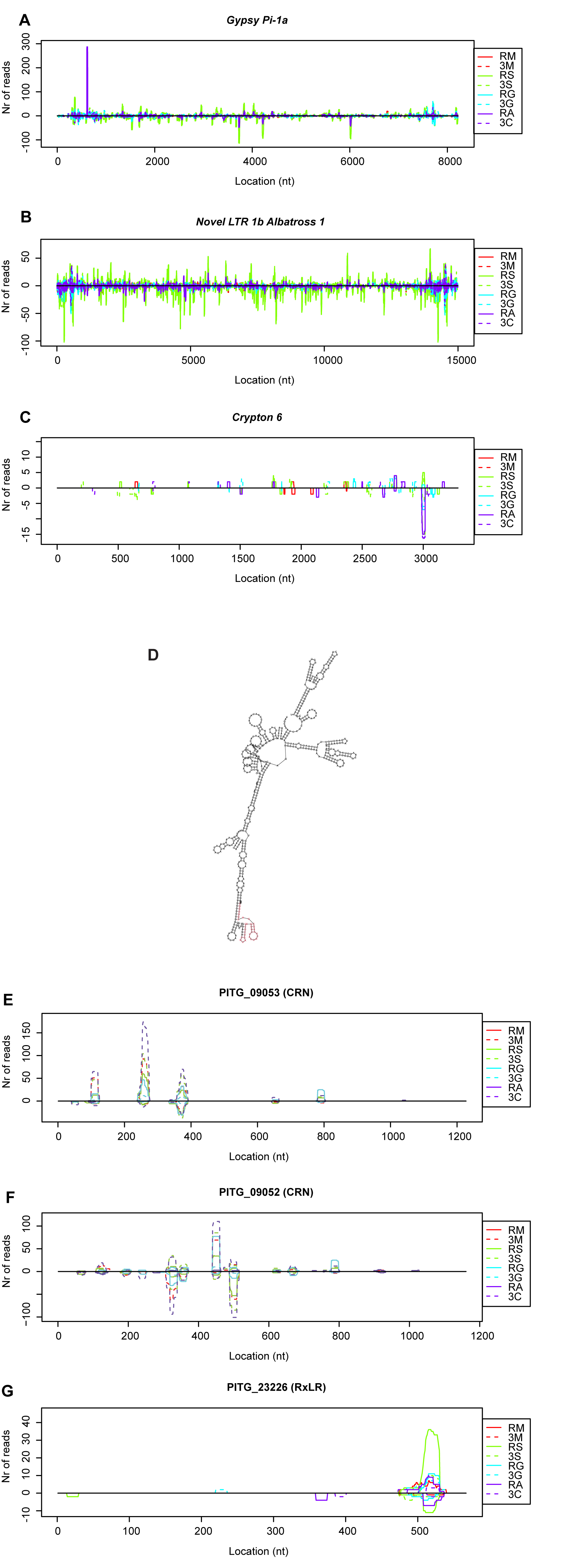

Supplement: Figure S5 — Distribution of sRNAs mapped to transposons, RxLR and CRN effector genes in different life cycle stages of P. infestans isolates R0 and 3928A, and specific sRNAs mapped to the predicted secondary structure (mfold 2.3) of Crypton6. A. Gypsy Pi-1a, B. Novel LTR 1b Albatross 1, C. Crypton6, D. Mapping of antisense sRNA to the 3′ end of Crypton6, indicated in red in the secondary structure, E. PITG_09053 (CRN), F. PITG_09052 (CRN), G. PITG_23226 (RxLR). R = isolate R0, and 3 = isolate 3928a; M = mycelium stage, S = sporangium, G = germinating sporangium, C = germinating cysts. The Crypton6 structure shown in D is the most optimal, with the lowest free energy. (TIF) [file pone.0051399.s005.tif]

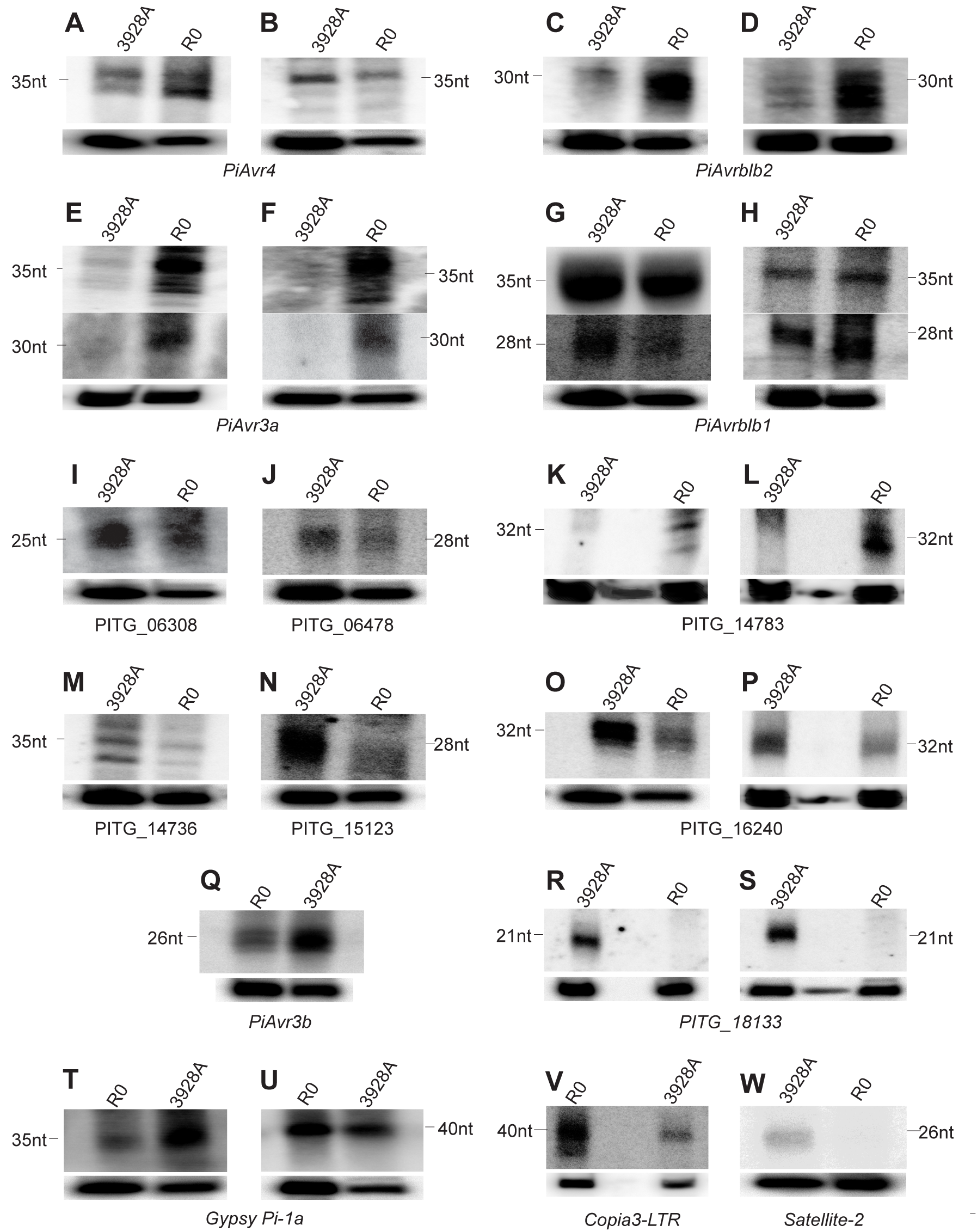

Supplement: Figure S6 — Northern hybridizations detecting sRNAs derived from RxLR and CRN effector genes, and transposons in P. infestans isolates R0 and 3928A. A–B. PiAvr4 (AS, S), C–D. PiAvrblb2 (S, AS), E–F. PiAvr3a (S, AS), G–H. PiAvrblb1 (S, AS), I. PITG_06308 (S), J. PITG_06478 (AS), K–L. PITG_14783 (S, AS), M. PITG_14736 (AS), N. PITG_15123 (S), O–P. PITG_16240 (S, AS), Q. PiAvr3b (AS), R–S. PITG_18133 (CRN; AS, S), T–U. Gypsy Pi-1a (AS, S), V. Copia3-LTR (AS), W. Satellite-2 (AS). S - sense strand. AS - antisense strand. Loading controls (U4 spliceosomal RNA) are shown below each autoradiograph. (TIF) [file pone.0051399.s006.tif]

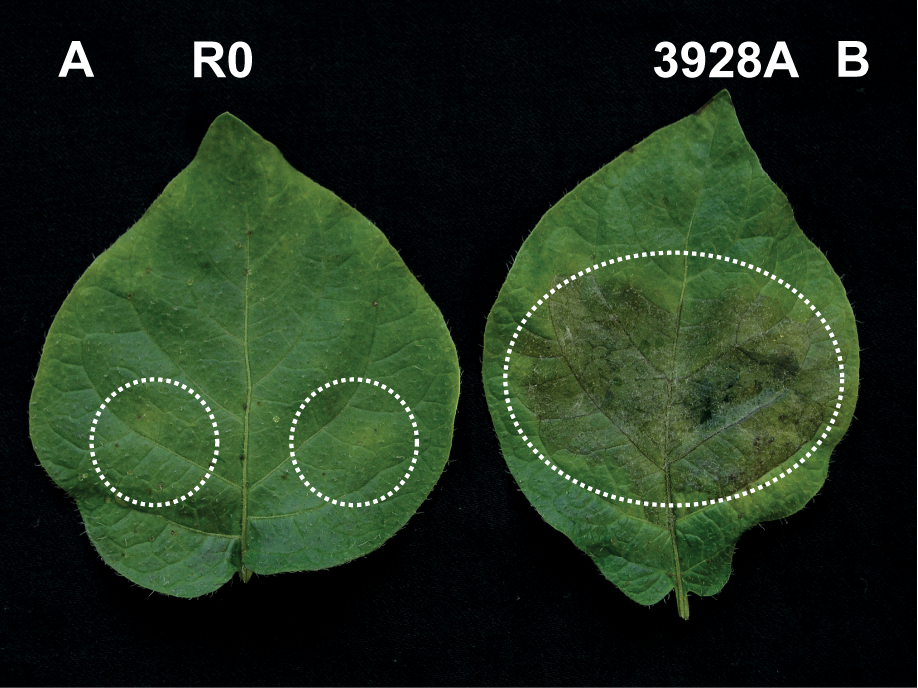

Supplement: Figure S7 — Comparison of the pathogenicity of isolates R0 and 3928A during infection of detached leaflets of potato cv. Bintje. R0 exhibited minimal or no disease (A, small circles), while 3928A formed a large sporulating disease lesion (B). Leaves were photographed 5 days after inoculation. (TIF) [file pone.0051399.s007.tif]

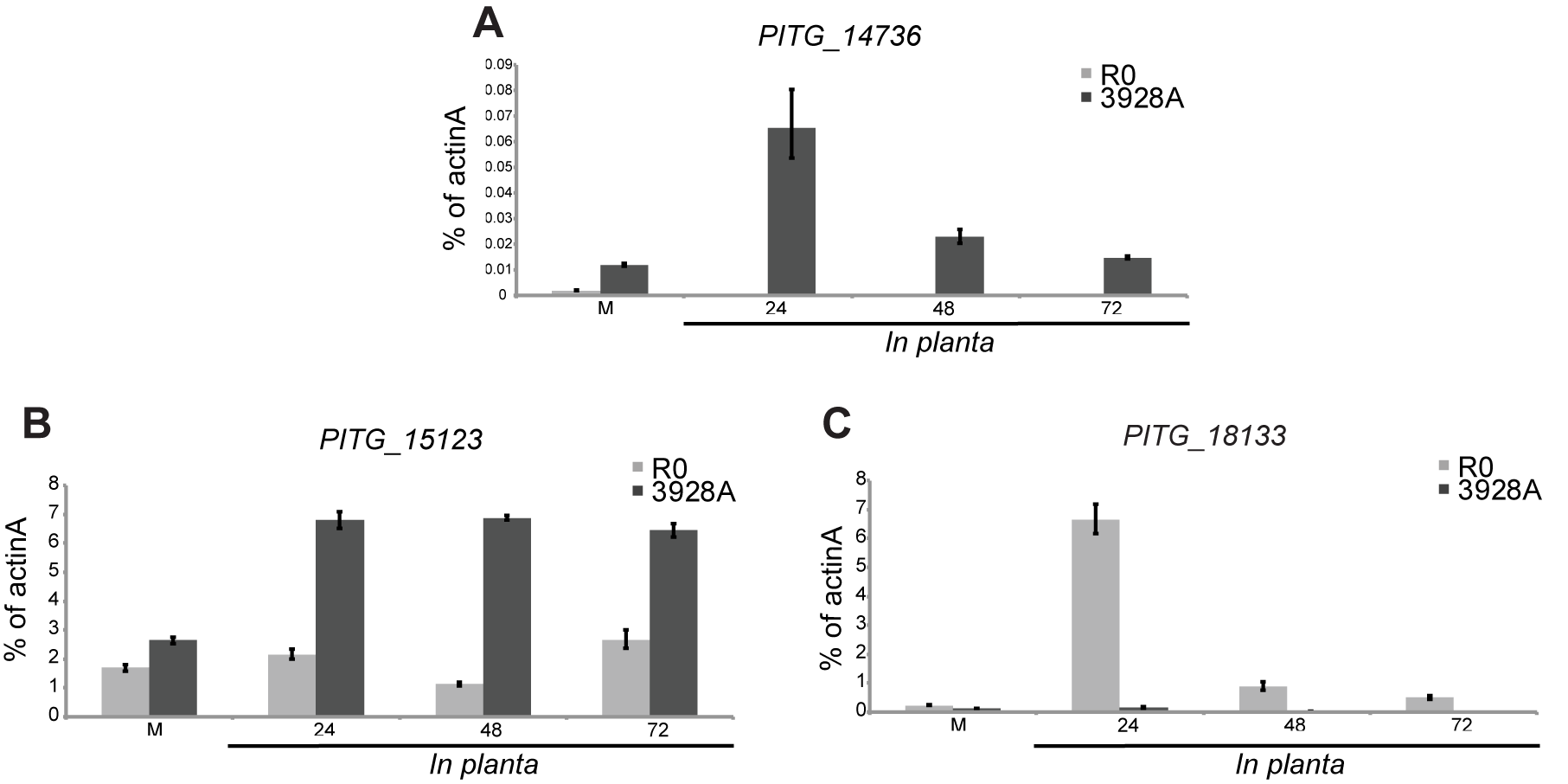

Supplement: Figure S8 — Relative transcript abundance (qRT-PCR) of RxLR and CRN effector genes at different infection time points in P. infestans isolates R0 and 3928A. A. PITG_14736 (RxLR), B. PITG_15123 (RxLR), C. PITG_18133 (CRN). The transcript profiles are shown at 24, 48 and 72 h post-inoculation on potato cultivar Bintje (no known resistance genes) relative to the mRNA level in cultured non-sporulating mycelium (M). In each graph, the light grey bar represents R0, and the dark bar represents 3928A. All calculations and statistical analyses were carried out as described in [90]. Error bars represent confidence intervals calculated using three technical replicates for each sample within the qRT-PCR assay. The abundance of mRNA for each gene is shown as a proportion of the actin A (PiactA) transcript on the y-axis of each graph. Amplifications repeated on independent occasions with different starting RNA and cDNA samples resulted in similar transcript accumulation profiles for all genes tested. (TIF) [file pone.0051399.s008.tif]

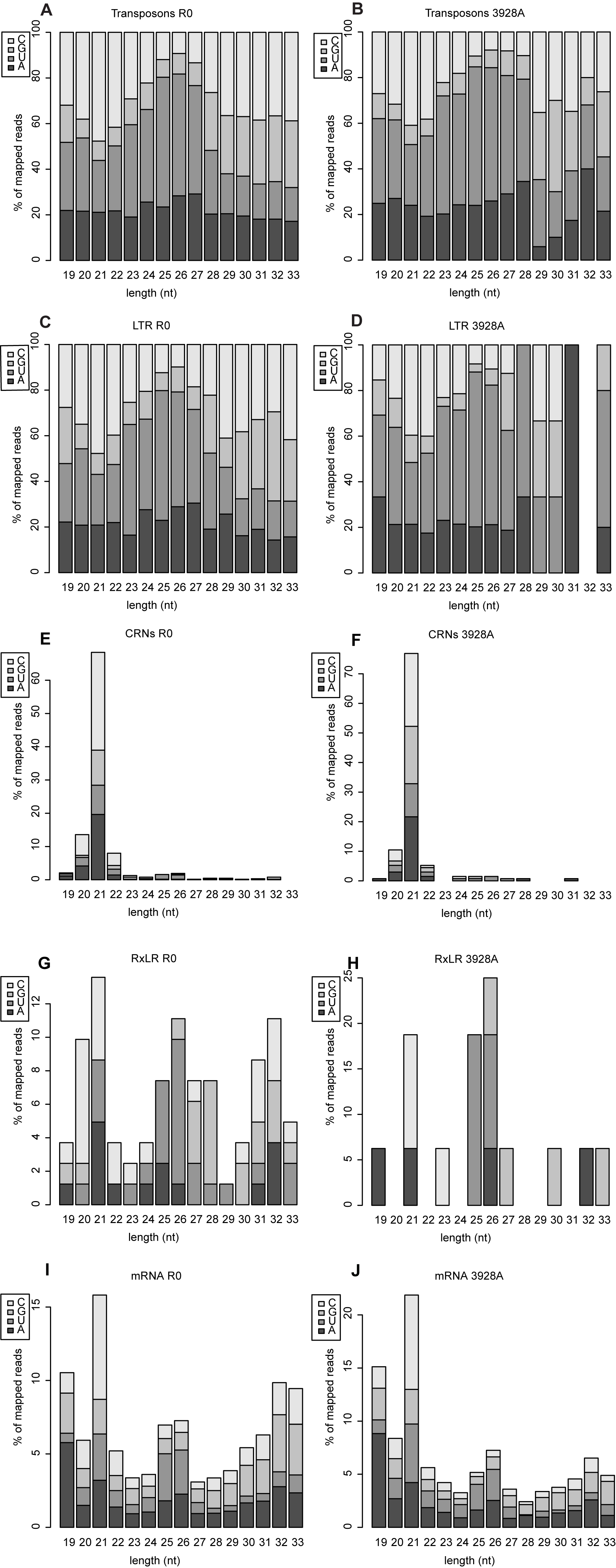

Supplement: Figure S9 — 5′ nucleotide preferences of sRNAs in P. infestans isolates R0 and 3928A mapped to: A–B. All transposons, C–D. LTR retrotransposon subset, E–F. CRN effector encoding genes, G–H. RxLR effector encoding genes, I–J. All predicted mRNAs (includes RxLR and CRN effector genes). Empty columns in a panel indicate that no sRNAs of the marked size were identified. (TIF) [file pone.0051399.s009.tif]

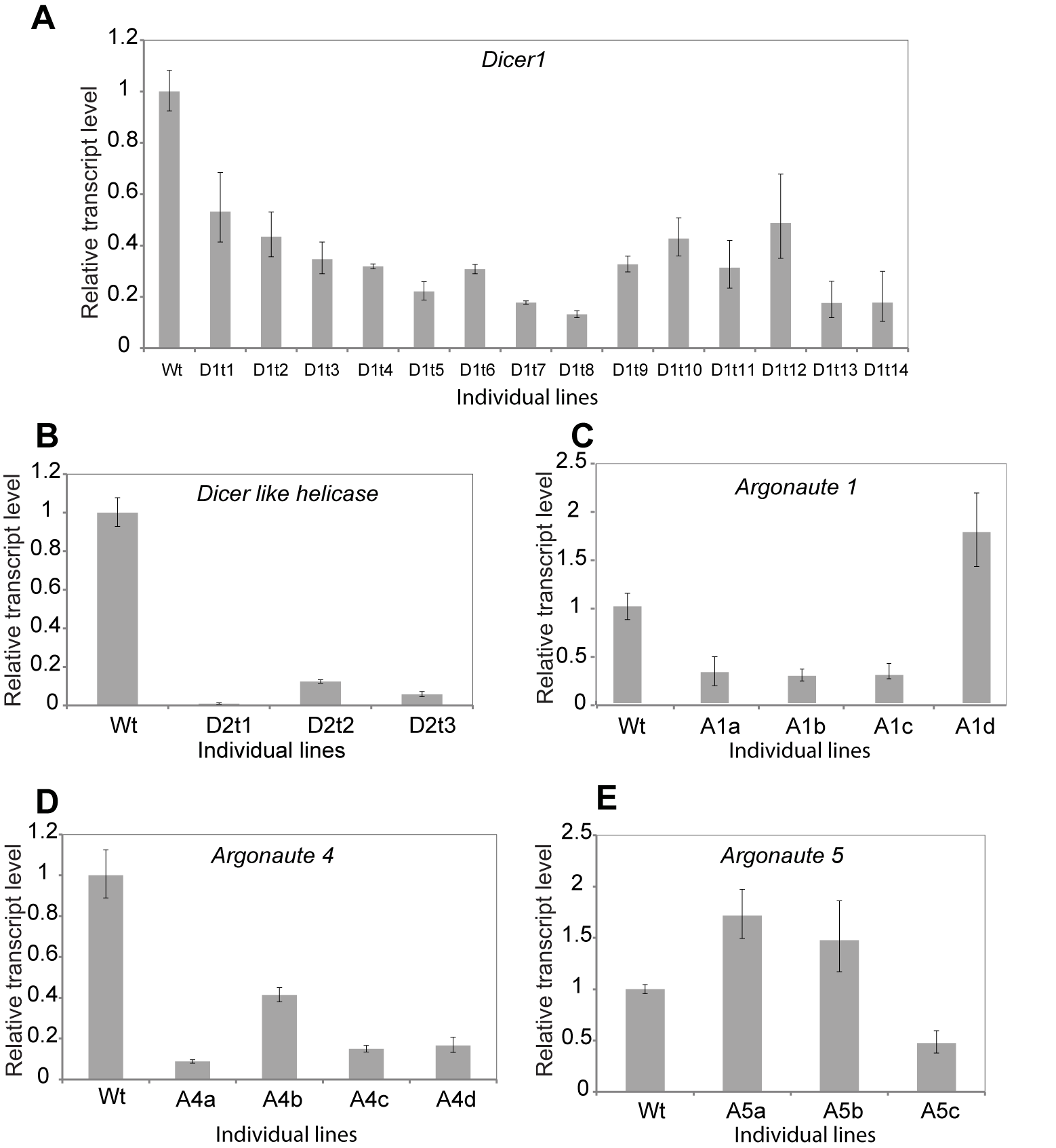

Supplement: Figure S10 — Silencing of genes encoding components of the silencing pathway in P. infestans. Comparison of PiDcl1 (dicer-like) and PiRnh5 (dicer-like helicase), PiAgo (Argonaute1, 4, 5) transcript abundance (qRT-PCR), individually, in wild type (88069; wt) and inverted repeat silenced lines. A. D1t1–D1t14 (PiDcl1), B. D2t1–D2t3 (PiRnh5), C. A1a–A1d (PiAgo1), D. A4a–A4d (PiAgo4), E. A5a–A5c (PiAgo5). All calculations and statistical analyses were carried out as described in [90]. Error bars represent confidence intervals calculated using three technical replicates for each sample within the RT-PCR assay. (TIF) [file pone.0051399.s010.tif]
