# Supplementary material for: Evidence for Small RNAs Homologous to Effector-Encoding Genes and Transposable Elements in the Oomycete Phytophthora infestans
Source: PLoS One. 2012 Dec 14;7(12):e51399. doi: 10.1371/journal.pone.0051399 (PMC3522703; doi:10.1371/journal.pone.0051399)
Supplement: Table S1 — Distribution of sRNA reads. (DOCX) [file pone.0051399.s011.docx]

**Table S1** Distribution of sRNA reads

|  | | | | | | | | |
| --- | --- | --- | --- | --- | --- | --- | --- | --- |
| Description | R0-M | R0-S | R0-GS | R0-GC | 3928a-M | 3928a-S | 3928A-GS | 3928A-GC |
| Total no. of reads generated | 2141084 | 6150060 | 3029238 | 3952194 | 435193 | 5456973 | 3602205 | 3319363 |
| After filtering sRNAs mapped to tRNA and rRNA | 2066569 | 5906230 | 2941924 | 3834109 | 427591 | 5363456 | 3524257 | 3187031 |
| 19 to 33nt size reads after filtering | 547526 | 1938368 | 906157 | 1099458 | 115166 | 1603474 | 1071665 | 681241 |
| Genome (Core Set) | 85502 | 213825 | 98001 | 109603 | 7585 | 124486 | 85526 | 56278 |
| All Transposons | 39465 | 186667 | 56394 | 59612 | 5414 | 91291 | 65653 | 37265 |
| Unique reference transposable elements | 11934 | 64796 | 17888 | 20607 | 1516 | 28616 | 20573 | 10916 |
| RxLR | 81 | 560 | 138 | 182 | 16 | 217 | 157 | 106 |
| CRN | 626 | 318 | 689 | 114 | 134 | 1811 | 1185 | 1046 |
| mRNA | 12394 | 49455 | 15457 | 15945 | 1779 | 19813 | 15834 | 12633 |
| Unassembled Genome | 76926 | 294248 | 109618 | 187169 | 7093 | 144139 | 89776 | 61285 |

GC- germinating cysts; GS- germinating sporangium; M-mycelium; S-sporangium
