# Supplementary material for: Evidence for Small RNAs Homologous to Effector-Encoding Genes and Transposable Elements in the Oomycete Phytophthora infestans
Source: PLoS One. 2012 Dec 14;7(12):e51399. doi: 10.1371/journal.pone.0051399 (PMC3522703; doi:10.1371/journal.pone.0051399)
Supplement: Table S3 — Predicted miRNA sequences and the location of their origin. (DOCX) [file pone.0051399.s013.docx]

**Table S3** Predicted miRNA sequences and the genomic location of their origin

| **Name** | **miRNA candidates** | **Length in nt** | **Genomic location** |
| --- | --- | --- | --- |
|  |  |  |  |
| PimiRNA1 | UUACACGCGUACAGCUAACCGUCA | 24 | 50 bp upstream *PITG_00222* |
| PimiRNA2 | ACGUUAAGUCAUAGUAACGAG | 21 | *PITG_12887* first exon |
| PimiRNA3 | UCUGGUCAAGCUUGUUUGAGUGUG | 24 | Intergenic |
| PimiRNA4 | CGUCAUGGUGCCGCCUGAACCAA | 23 | Intergenic |
| PimiRNA5 | AUCGGGCAACAGCUGUCCGUAU | 22 | 500 bp downstream *PITG_17805* |
| PimiRNA6 | UGGCUCGAUCUACGGUGCAGU | 22 | *PITG_00907* first exon |
