# Supplementary material for: Evidence for Small RNAs Homologous to Effector-Encoding Genes and Transposable Elements in the Oomycete Phytophthora infestans
Source: PLoS One. 2012 Dec 14;7(12):e51399. doi: 10.1371/journal.pone.0051399 (PMC3522703; doi:10.1371/journal.pone.0051399)
Supplement: Table S5 — List of oligonucleotide primers used for riboprobes, DNA oligonucleotide probes, qRT-PCR, and DNA cloning. (DOCX) [file pone.0051399.s015.docx]

**Table S5** List of oligonucleotide primers used for riboprobes, DNA oligonucleotide probes, qRT-PCR, and DNA cloning

| **Gene locus and/or name** | **Primer name** | **Sequence** |
| --- | --- | --- |
|  |  | **Riboprobe generation** |
| *PITG_21388*  (*PiAvrblb1*) | 21388 -FW  21388-RV | ATATACTAGTCTCCTGTTGACCGTGCTTTT  ATATGCGGCCGCCTAGCTAGGGCCAACGTTTTT |
| *PITG_07387*  (*PiAvr4*) | 07387-FW  07387-RV | ATATACTAGTATGCGTTCGCTTCACATTTT  ATATGCGGCCGCGGCCGTCTAGCTTGGAGAC |
| *PITG_20300/20303*  (*PiAvrblb2*) | 20300-FW  20300-RV | ATATACTAGT ATGCGCAGTTTTCTCTACGG  ATATGCGGCCGCCTTCGTCATTTTTGCTTTTGC |
| *PITG_14371*  (*PiAvr3a*) | 14371-FW  14371-RV | ATATACTAGTCGTCTGGCAATTATGCTGTCT  ATATGCGGCCGCCAGGTGCATCAGGTAGCTATTG |
| *PITG_06308* | 06308-FW  06308-RV | ATATACTAGTTTGATCATTTTCGTCGCTTTC  ATATGCGGCCGCTCAAAGAGCACGACATACTCC |
| *PITG_06478* | 06478S-FW  06478S-RV  06478AS-FW  06478AS-RV | TAATACGACTCACTATAGGGAGACTGCTTATGGCTGCCTTCTT  CCGATAGGGTTGTCGTTGTC  CTGCTTATGGCTGCCTTCTT  TAATACGACTCACTATAGGGAGACCGATAGGGTTGTCGTTGTC |
| *PITG_14783* | 14783-FW  14783-RV | ATATACTAGTGCGTCTTCCTTACGTGTTCG  ATATGCGGCCGCGGTGGCTTCTTCTTCTTGTTTT |
| *PITG_15123* | 15123-FW  15123-RV | ATATACTAGTCTGTGTGCGATTGCAACTTT  ATATGCGGCCGCTGAGGAGGAGCCATCTTTTT |
| *PITG_16240* | 16240-FW  16240-RV | ATATACTAGTCTGTGTTTGGCGCTGATG  ATATGCGGCCGCTTCCACCACTTCTTCGACTTC |
| *PITG_18133* | 18133-FW  18133-RV | ATATACTAGTGGGTATGGCGAGAAGTGTGT  ATATGCGGCCGCTCAAGTGACCACCGAATGTG |
| *PITG_18215*  (*PiAvr3b*) | 18215-FW  18215-RV | ATATACTAGTACTGCGATCCTGACCTATGG  ATATGCGGCCGCCTTTGCGGTCAGGACGTTAT |
| *PITG_14736* | 14736S-FW  14736S-RV  14736AS-FW  14736AS-RV | TAATACGACTCACTATAGGGAGAGCGTCTGTCCTGCGTCTATC  GCGGTACAGCATACGGAGAT  GCGTCTGTCCTGCGTCTATC  TAATACGACTCACTATAGGGAGAGCGGTACAGCATACGGAGAT |
| *Gypsy Pi-1a* | GypsyPi11-FW  GypsyPi11-RV  GypsyPi12-FW  GypsyPi12-RV  GypsyPi13-FW  GypsyPi13-RV  GypsyPi14-FW  GypsyPi14-RV | ATATACTAGTTGTAACGGGGTACGTAGCC  ATATGCGGCCGCGTGGGAACATGACTCACCAA  ATATACTAGTCTCTGTCAGGACCCGGGG  ATATGCGGCCGCTATTTCATTGCCAGGAGCTCC  ATATACTAGTTGCCCTTGTGAAGTTCCG  ATATGCGGCCGCGGAATCAGGGCGAGCTCG  ATATACTAGTAGCATCACCGCCAGTCCC  ATATGCGGCCGCTGTGACGGGGTCACACAG |
| *Satellite-2* | Satellite2S-FW  Satellite2S-RV | TAATACGACTCACTATAGGGAGAGATTTGAGAAGCAGGCAAGC  GGGCTAAGGCCGTATCTACC |
|  |  | **DNA oligonucleotide probes** |
| *PITG_22969* |  | AAGGACTTGTGCACCGTGGATCCTG |
| *PITG_21388* |  | ACGTGTTGGGCTCTTTTGTGACCT |
| *Copia3-LTR-21* |  | CCGGTCTCCGCACCAAGTGCA |
| *Crypton-6* |  | CTACGGCTGAGCTACGGGGGC |
| *Copia3-LTR-40* |  | GATCCCGCTGTGATTATGGTA |
| *U4-snRNA* |  | GAGTAGTTTTCAACTAGCAAT |
|  |  | **qRT-PCR** |
| *PITG_14371*  (*PiAvr3a*) | Avr3a TAQF  Avr3a TAQR | CGCCATAAACTTTGCAACCA  TGCCGGCTGAATCGTGTAT |
| *PITG_18215*  (*PiAvr3b*) | Avr3b-QFW  Avr3b-QRV | CATCAGAACTGGGACGCTCT  GGAGTACGCTCTCAGCCATC |
| *PITG_16663*  (*PiAvr1*) | qRTPCR_16663bFo  qRTPCR_16663bRe | GAGCAAGATCGACGAGTTCA  CCTCAGGTGATCCTCCACTT |
| *PITG_06308* | qNud-f  qNud-r | TCTCCGACCCAACAAGCATT  TCGCCCCTCTGTCTTCACCT |
| *PITG_21388*  (*PiAvrBlb1*) | QrtIpiO1_For  QrtIpiO1_Rev | TGCGTTCGCTCCTGTTGA  CGGTGTTGAGATTGGATGAAAC |
| *PITG_20303*  (*PiAvrBlb2*) | QrtBlb2Fam_For  QrtBlb2Fam_Rev | CGAGTCTCGCCCCTTGT  GTTTTTCCAGAACCCACCAT |
| *PITG_07387*  (*PiAvr4*) | qRTPITG_07387Fo  qRTPITG_07387Re | GCGCTCCTGAAGTATGTCAA  TCTAGCTTGGAGACGGGATT |
| *PITG_23226* | 23226-QFW  23226-QRV | GGACAGTTCAAGACCGAAGC  GTCACTCCGCCCACTACTGT |
| *PITG_15117*  (*PiActA*) | ActAF  ActAR | CATCAAGGAGAAGCTGACGTACA  GACGACTCGGCGGCAG |
| *PITG_14783* | 14783RT-FW  14783RT-RV | ATTTGAAGCGGGGAATTCTT  TACTCGTCCGTTTGCATGAG |
| *PITG_14736* | 14736RT-FW  14736RT.RV | CATCGCAAGCGCTAATGCT  GTGAACTGAAACTTCTGGTGATGC |
| *PITG_15123* | 15123RT-FW  15123RT-RV | GGAGAAGAGCAACTGGCAAC  CAATTCCACTCGAACCCACT |
| *PITG_18133* | 18133RT-FW  18133RT-RV | GGTGGCTGACTTGAAGAAGG  GAGAAGGGAGCCACTTGTTG |
| *PITG_22969* | 22969RT-FW  22969RT-RV | CGATCAAGGCGAAGAAGAAG  CAAGCGAGAAGAGAGCCACT |
|  |  | **Cloning of hairpin constructs** |
| *PITG_09292*  (*PiDcl1*) | DicerA-FW  DicerA-RV  DicerB-FW  DicerB-RV | ATATGGCCGGCCTTCGCAACGACAGACTCAAC  ATATCCTGCAGGGTGACTGTTGCCACCATTTG  ATATCCGCGGTTCGCAACGACAGACTCAAC  ATATGGCGCGCCGTGACTGTTGCCACCATTTG |
| *PITG_09951*  (*PiRnh5*) | Rnh5A-FW  Rnh5A-RV  Rnh5B-FW  Rnh5B-RV | ATATGGCCGGCCGTGTCGCTTAGTCGCATGAA  ATATCCTGCAGGCTCGGTTCTGTTCTCCTTCG  ATATCCGCGGGTGTCGCTTAGTCGCATGAA  ATATGGCGCGCCCTCGGTTCTGTTCTCCTTCG |
| *PITG_04470*  (*PiAgo1*) | Ago1A-FW  Ago1A-RV  Ago1B-FW  Ago1B-RV | ATATGGCCGGCCAAGACTCCGAGCCCGTTATT  ATATCCTGCAGGCTCCTCTACGAAGGCAGTGG  ATATCCGCGGAAGACTCCGAGCCCGTTATT  ATATGGCGCGCCCTCCTCTACGAAGGCAGTGG |
| *PITG_01400*  (*PiAgo3*) | Ago3A-FW  Ago3A-RV  Ago3B-FW  Ago3B-RV | ATATGGCCGGCCTTACGATGATCGCAGCAGTC  ATATCCTGCAGGACACAGGGTCGCCAATACTC  ATATGGCGCGCCTTACGATGATCGCAGCAGTC  ATATGGCGCGCCACACAGGGTCGCCAATACTC |
| *PITG_01443*  (*PiAgo4*) | Ago4A-FW  Ago4A-RV  Ago4B-FW  Ago4B-RV | ATATGGCCGGCCGAGTGGTGGTGGACGCTAAT  ATATCCTGCAGGTAGAGCTCGCATTTCGGTTT  ATATCCGCGGGAGTGGTGGTGGACGCTAAT  ATATGGCGCGCCTAGAGCTCGCATTTCGGTTT |
| *PITG_01444*  (*PiAgo5*) | Ago5A-FW  Ago5A-RV  Ago5B-FW  Ago5B-RV | ATATGGCCGGCCCTGCCAATATGACGATGGTG  ATATCCTGCAGGCAGACATGCGCTTCACATCT  ATATCCGCGGCTGCCAATATGACGATGGTG  ATATGGCGCGCCCAGACATGCGCTTCACATCT |
